# Rapid processing and quantitative evaluation of structural brain scans for adaptive multimodal imaging

**DOI:** 10.1002/hbm.25755

**Published:** 2021-12-24

**Authors:** František Váša, Harriet Hobday, Ryan A. Stanyard, Richard E. Daws, Vincent Giampietro, Owen O'Daly, David J. Lythgoe, Jakob Seidlitz, Stefan Skare, Steven C. R. Williams, Andre F. Marquand, Robert Leech, James H. Cole

**Affiliations:** ^1^ Department of Neuroimaging Institute of Psychiatry, Psychology & Neuroscience, King's College London London UK; ^2^ Department of Forensic & Developmental Sciences Institute of Psychiatry, Psychology & Neuroscience, King's College London London UK; ^3^ The Computational, Cognitive and Clinical Neuroimaging Laboratory, Department of Brain Sciences Imperial College London London UK; ^4^ Department of Child and Adolescent Psychiatry and Behavioral Science Children's Hospital of Philadelphia Philadelphia Pennsylvania USA; ^5^ Department of Psychiatry University of Pennsylvania Philadelphia Pennsylvania USA; ^6^ Department of Neuroradiology Karolinska University Hospital Stockholm Sweden; ^7^ Department of Clinical Neuroscience Karolinska Institutet Stockholm Sweden; ^8^ Donders Institute for Brain, Cognition and Behavior Radboud University Nijmegen Nijmegen The Netherlands; ^9^ Department for Cognitive Neuroscience Radboud University Medical Center Nijmegen Nijmegen The Netherlands; ^10^ Department of Computer Science, Centre for Medical Image Computing University College London London UK; ^11^ Dementia Research Centre, Institute of Neurology University College London London UK

**Keywords:** EPImix, fingerprinting, identifiability, morphometric similarity, MRI, reliability, structural covariance

## Abstract

Current neuroimaging acquisition and processing approaches tend to be optimised for quality rather than speed. However, rapid acquisition and processing of neuroimaging data can lead to novel neuroimaging paradigms, such as adaptive acquisition, where rapidly processed data is used to inform subsequent image acquisition steps. Here we first evaluate the impact of several processing steps on the processing time and quality of registration of manually labelled T_1_‐weighted MRI scans. Subsequently, we apply the selected rapid processing pipeline both to rapidly acquired multicontrast EPImix scans of 95 participants (which include T_1_‐FLAIR, T_2_, T_2_*, T_2_‐FLAIR, DWI and ADC contrasts, acquired in ~1 min), as well as to slower, more standard single‐contrast T_1_‐weighted scans of a subset of 66 participants. We quantify the correspondence between EPImix T_1_‐FLAIR and single‐contrast T_1_‐weighted scans, using correlations between voxels and regions of interest across participants, measures of within‐ and between‐participant identifiability as well as regional structural covariance networks. Furthermore, we explore the use of EPImix for the rapid construction of morphometric similarity networks. Finally, we quantify the reliability of EPImix‐derived data using test–retest scans of 10 participants. Our results demonstrate that quantitative information can be derived from a neuroimaging scan acquired and processed within minutes, which could further be used to implement adaptive multimodal imaging and tailor neuroimaging examinations to individual patients.

## Graphical abstract

We first evaluate the impact of several processing steps on the processing time and quality of registration of manually labelled T_1_‐weighted MRI scans, and apply the selected rapid processing pipeline both to standard T1‐weighted scans, as well as rapidly acquired multicontrast EPImix scans (which include T_1_‐FLAIR, T_2_, T_2_*, T_2_‐FLAIR, DWI and ADC contrasts, acquired in ~1 min). We then quantify the correspondence between rapidly processed EPImix T_1_‐FLAIR and single‐contrast T_1_‐weighted scans using several methods, and explore the use of EPImix for the rapid construction of morphometric similarity networks. Our results demonstrate that quantitative information can be derived from a neuroimaging scan within minutes; this could be used to implement adaptive multimodal imaging, where rapidly processed data is used to inform subsequent image acquisition steps and tailor neuroimaging examinations to individual patients.

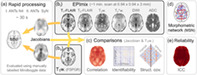

## 1 INTRODUCTION

An MRI scanner can be used to acquire a range of different contrasts, which provide complementary information and are sensitive to different pathophysiologies (Cercignani & Bouyagoub, 2018). Currently, multimodal MRI scanning involves specifying a sequence of contrasts prior to data acquisition and in research contexts, acquiring the same sequence for each individual. In a clinical context, the selection of contrasts is guided by factors such as clinical history, cognitive and neurological examinations, and symptoms (Camprodon & Stern, 2013). However, the optimal sequence of contrasts and/or parameters for each contrast may depend on the anatomical or physiological abnormalities specific to the individual patient or be specific to a given pathology, and thus may not be known a priori. As an alternative approach, it was recently proposed that data could be analysed as it is being acquired, with the near‐real‐time results used to determine subsequent acquisition steps (Cole et al., 2019). This approach was illustrated using three simulated scenarios, including (a) tailoring the resolution and/or field of view (FoV) of a structural scan to detect stroke, (b) adaptively acquiring multimodal data to classify a known outcome variable using a decision tree, and (c) adaptively searching across multiple MRI modalities using Bayesian optimisation to detect abnormality. However, adaptive acquisition is yet to be implemented practically. One prerequisite to progress beyond simulated scenarios (Cole et al., 2019) and implement adaptive acquisition in practice is the development of rapid analysis pipelines for multiple MRI modalities, enabling data to be processed in near‐real‐time.

We propose to capitalise on EPImix—a recently developed multicontrast sequence which acquires six contrasts (T_1_‐FLAIR, T_2_, T_2_*, T_2_‐FLAIR, DWI, ADC), at 0.975 × 0.975 × 3 mm resolution, in ~1 min (Skare et al., 2018). A multicontrast sequence such as EPImix, or other similar rapid multicontrast sequences (Polak et al., 2020), is well suited to be the first sequence in an adaptive acquisition run, rapidly providing an overview of neuroanatomy across multiple contrasts. EPImix contrasts have previously been compared to high‐quality, single‐contrast sequences to evaluate their suitability for qualitative disease identification and categorisation by trained radiologists, and have shown comparable diagnostic performance to routine clinical brain MRI (Delgado et al., 2019; Ryu et al., 2020). However, there have been no quantitative comparisons of EPImix and corresponding single‐contrast scans.

**FIGURE 1:**
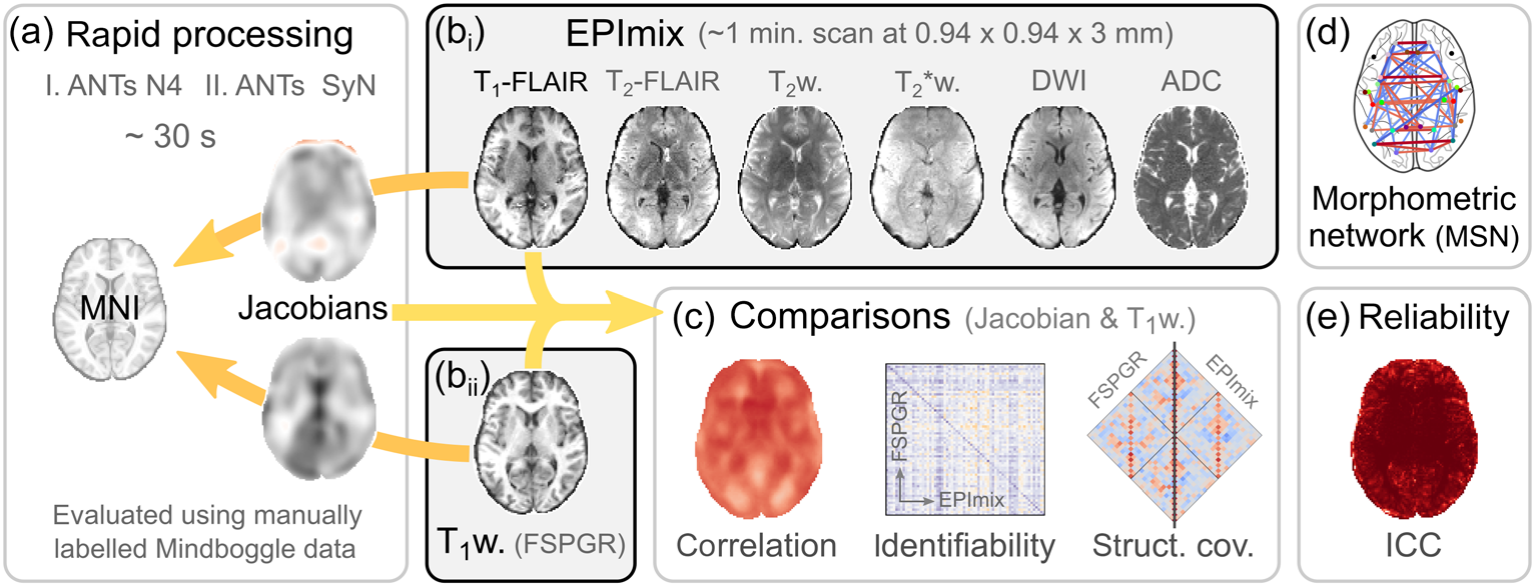
Overview of analysis steps. (a) A rapid processing pipeline for T_1_‐w scans was evaluated using the manually labelled Mindboggle dataset (Klein & Tourville, 2012; for details, see Figure 2). (b) The pipeline was used to process T_1_‐FLAIR scans derived from the rapid multicontrast EPImix sequence (Skare et al., 2018) as well as single‐contrast (IR‐FSPGR) T_1_‐w scans. (c) Jacobian determinants and tissue intensities derived from both types of T_1_‐w scan were compared using several methods, including correlation (across participants), inter‐individual identifiability, and structural covariance networks. (d) Additionally, we explored using the EPImix sequence to construct morphometric similarity networks (MSNs; Seidlitz et al., 2018). (e) Finally, we evaluated the test–retest reliability of all contrasts within the EPImix sequence, and of the derived MSNs

Here, we explore rapid image processing pipelines for the EPImix sequence, as well as for a single‐contrast T_1_‐weighted (T_1_‐w) sequence, and use the rapidly processed scans to quantitatively compare EPImix and standard T_1_‐w scans (Figure 1c). We first optimise a rapid processing pipeline by evaluating the impact of several processing steps on the processing time and on the quality of registration of manually labelled scans, using openly available data with manual segmentations in both native and standard space (Klein & Tourville, 2012). Subsequently, we quantify, in several ways, the overlap between selected EPImix contrasts and corresponding single‐contrast sequences. Finally, we demonstrate a novel quantitative application of the multicontrast EPImix sequence, which could be useful both in an adaptive imaging paradigm and beyond: the construction of morphometric similarity networks (MSNs; Seidlitz et al., 2018).

## 2 METHODS

### 2.1 Processing steps

While developing a rapid image processing pipeline, we considered the following factors to guide selection of steps:

- *Speed*: Faster processing was preferred. We measured speed in seconds. (Processing was run on an Apple Macbook Pro [2.2 GHz Intel Core i7, 16 Gb 1,600 MHz DDR3 RAM], with no other user processes running in parallel).
- *Quality*: Higher quality was preferred. We evaluated the quality of steps up to and including registration by quantifying overlap between source and target of manually labelled atlases (Klein et al., 2009) using the Mindboggle dataset (Klein & Tourville, 2012).
- *Automation*: Fewer quality control steps and resulting re‐running of processing steps following manual interventions and/or changes of parameters were preferred.

For the processing steps considered for inclusion in the pipeline, see Table 1.

**TABLE 1:** Processing steps considered

Step	Reason	Options	*Algorithm* (reference[s])
Downsampling	To save time (and potentially help extraction)	1/2/3 mm	ANTs *ResampleImageBySpacing* (Avants et al., 2011; Avants, Epstein, Grossman, & Gee, 2008)
Bias field correction	Commonly applied to improve registration	Yes/no	ANTs *N4BiasFieldCorrection* (Tustison et al., 2010)
Brain extraction	To improve registration	Yes/no	FSL *BET* (Smith, 2002)
Registration	To evaluate deviation from spatially normalised group	SyN/b‐spline SyN	ANTs *antsRegistrationSyNQuick.sh* (Avants et al., 2008, 2011)
Smoothing	To remove noise in voxel‐wise analyses	2/4/6 mm FWHM	Python *nilearn nl.image.smooth_img* (Abraham, Pedregosa, Eickenberg, & Gervais, 2014)

*Note*: For each step, we list the reason for consideration, the evaluated options and the algorithm used, including relevant references.

For registration of scans to standard space, we used ANTs (Avants et al., 2008), due to its good performance in systematic evaluations of registration algorithms (Bartel et al., 2019; Klein et al., 2009; Nazib, Galloway, Fookes, & Perrin, 2018). We are aware that the combination of processing steps listed in Table 1 is by no means exhaustive, as different software suites could have been used for each step, potentially differing in speed and quality of processing; instead, the selected steps serve as a proof‐of‐principle evaluation of the proposed approach (see also Section 4).

### 2.2 Evaluating the speed and quality of registrations

We evaluated the quality of registrations as well as the effect of any prior pre‐processing steps using the Mindboggle dataset (Klein & Tourville, 2012), which contains T_1_‐w scans of 101 healthy participants manually labelled according to the Desikan–Killiany–Tourville (DKT) protocol (31 cortical regions per hemisphere). The dataset contains both T_1_‐w scans and manual DKT atlas labels in both native and MNI152 spaces. These manual labels have previously been used as a gold standard in evaluations of processing tools (e.g., Henschel et al., 2020; Tustison et al., 2014; Velasco‐Annis et al., 2017). We used the non‐skull‐stripped T_1_‐w scans as initial input into our processing pipelines as brain extraction is one of the processing steps under evaluation.

**FIGURE 2:**
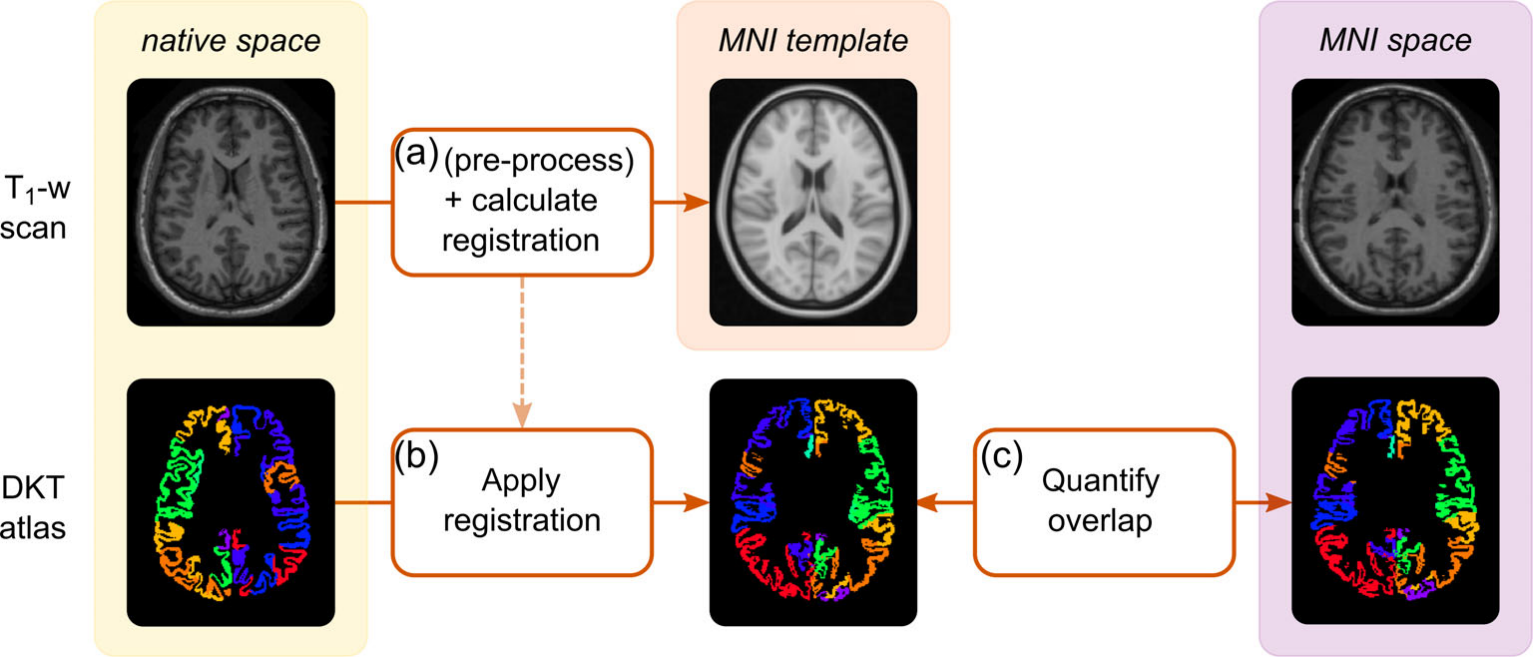
Using manual Desikan–Killiany–Tourville (DKT) atlas labels from the Mindboggle dataset to quantitatively evaluate the quality of registration (and pre‐processing steps). (a) The processing pipeline (up to and including registration) is applied to the native‐space T_1_‐w scan to transform it to MNI152 space and to estimate registration parameters. (b) The registration (calculated in step a) is applied to the native‐space DKT atlas. (c) The Dice coefficient is used to quantify the overlap, in MNI152 space, between the atlas labels which have been transformed from native space (in step b) and the manual atlas labels released with the Mindboggle dataset (Klein & Tourville, 2012)

We first used the native space T_1_‐w scan to estimate registration parameters to MNI152 space (following any optional pre‐processing steps; Figure 2a). Subsequently, we applied the registration step to the manual native space DKT atlas labels (Figure 2b). Finally, we quantified the overlap of the transformed atlas labels with the manual MNI152 space atlas labels using the Dice coefficient (Figure 2c), equal to twice the number of overlapping voxels divided by the sum of the number of voxels in each set; for voxel sets {A} and {B}:

(1) $$D=\frac{2\cdot |A\cap B|}{|A|+|B|}$$

We calculated the Dice coefficient both for all atlas regions across the brain at once, and for individual atlas regions.

We evaluated the above steps (Table 1) in a sequential manner, as follows. (As the options evaluated at each step depend on results obtained in the previous step, we report the outcome of each step here; for details underlying our selection, see Section 3. Unless otherwise specified, we used the ANTs SyN registration as implemented by default in *antsRegistrationSyNQuick.sh* as the main processing step.)

1. We first evaluated the effect of spatial resolution, including 1 mm (native Mindboggle data resolution), 2 mm, and 3 mm isotropic. We downsampled both the T_1_‐w scans and DKT atlases in both native and standard space, before applying the ANTs SyN algorithm for registration (Avants et al., 2008).
2. Following selection of the resolution (2 mm), we considered the effect of bias field correction. We compared the quality and speed of ANTs SyN registration with and without ANTs N4 bias field correction (Tustison et al., 2010).
3. We next considered the impact of brain extraction on the output of the previous steps (2 mm with bias field correction). We compared the default non‐skull‐stripped registration to the application of FSL BET (Smith, 2002) for skull‐stripping. (We used default BET parameters, except for the fractional intensity threshold, which was set to 0.4 based on an initial test evaluation using a subset of scans.)
4. Finally, we applied ANTs spline‐based SyN registration to the output of previous steps (2 mm with bias field correction and without skull‐stripping) to compare speed and quality to standard ANTs SyN (Avants et al., 2008).

+ Additionally, we evaluated a reference pipeline, optimised for quality rather than speed. This consisted of 1 mm isotropic resolution images, ANTs N4 bias field correction and the slower *antsRegistrationSyN.sh* script, optimised for quality.

As a quality control step, the T_1_‐w scan in MNI152 space (i.e., the output of Figure 2a) was visually assessed to ensure a successful registration. For details of the settings used for each processing step in each evaluated pipeline, see Table 2.

**TABLE 2:** Evaluated pipelines

Pipeline	Resolution	Bias field corr.	Brain extraction	Registration
1	1/2/3 mm	Off	Off	SyN
2	2 mm	On/off	Off	SyN
3	2 mm	On	On/off	SyN
4	2 mm	On	Off	SyN/b‐spline SyN
Final	2 mm	On	Off	SyN
+ Reference	1 mm	On	Off	‘slow’ SyN

*Note*: Settings used for each step while evaluating pipelines in a sequential manner. Cell colour indicates evaluation status: yellow cells indicate steps under evaluation, orange cells indicate steps not yet evaluated, and green cells indicate evaluated steps, where an option has been selected. (Steps within the reference pipeline were not evaluated sequentially.).

We note that not all combinations of processing steps were systematically evaluated. Moreover, our aim was not to find the ‘optimal’ processing pipeline, but rather to consider trade‐offs in processing speed and quality, to identify a combination of processing steps which optimises both parameters (i.e., ‘good enough and fast enough’). As we argue in further detail in Section 4, we deliberately avoided combining speed and quality into a single evaluation metric, as the relative importance of these two criteria cannot easily be quantified. Instead, we believe that these two quantitative measures should serve to guide the construction of a rapid processing pipeline on a case‐by‐case basis.

### 2.3 Processing of EPImix and corresponding single‐contrast T_1_ ‐w scans

Following the selection of a rapid processing pipeline (2 mm scans with bias field correction and standard SyN registration, see also Section 3), we applied it to the EPImix scans, and corresponding T_1_‐w single‐contrast scans. We focused on the T_1_‐w single‐contrast sequence due to data availability.

We included scans collected on the same scanner (General Electric MR750 3.0T, Waukesha, WI) across three different studies conducted on healthy volunteers at the Centre for Neuroimaging Sciences, King's College London's Institute of Psychiatry, Psychology & Neuroscience. The studies received ethical approval from King's College London's Psychiatry, Nursing and Midwifery Research Ethics Committee (KCL Ethics References: HR‐18/19‐9268, HR‐18/19‐11058, and HR‐19/20‐14585). All participants gave written informed consent to take part in the study.

EPImix scans were collected from 95 participants (48 female, 47 male; age median [first, third Quartile] (Md [Q_1_,Q_3_]) = 25 [22,29] years; Figure S1), consisting of six contrasts (T_2_*, T_2_‐FLAIR, T_2_, T_1_‐FLAIR, DWI, ADC) acquired at 0.975 × 0.975 × 3 mm resolution. For details regarding specific acquisition parameters, see Supporting Information. The EPImix sequence includes an on‐scanner motion correction step; the motion corrected images were used for further analyses. For further details regarding the EPImix sequence, see Skare et al. (2018). Additionally, for 10 participants, a second EPImix scan was acquired during the same session to investigate test–retest reliability.

Of the participants scanned with the EPImix sequence, 66 were additionally scanned, within the same session, with an IR‐FSPGR T_1_‐weighted sequence (33 female, 33 male; age Md [Q_1_,Q_3_] = 25 [23,29.75] years; Figure S1). Of these, 12 were scans acquired at 1 × 1 × 1 mm resolution, and 54 were scans acquired at 1.05 × 1.05 × 1.2 mm resolution. For details regarding specific acquisition parameters, see Supporting Information.

Note that as both the EPImix T_1_‐FLAIR contrast and the single‐contrast IR‐FSPGR sequence are T_1_‐weighted, we hereafter refer to both as such (as well as simply ‘T_1_‐w’).

When applying the previously identified rapid processing pipeline to EPImix scans, we omitted the downsampling step (to 2 mm isotropic resolution), as options for modifying the EPImix voxel resolution of 0.975 × 0.975 × 3 mm during acquisition are limited, and the ‘native’ EPImix resolution resulted in sufficiently rapid processing (Md [Q_1_,Q_3_] = 32 [31,33] s across participants; see also Figure 4). Instead, we registered EPImix T_1_‐w scans directly to a 2 mm isotropic MNI template, and subsequently applied the same transformation to the remaining EPImix contrasts. Furthermore, following registration of the single‐contrast and EPImix T_1_‐w scans to MNI space, we extracted the logarithm of the Jacobian determinant of the ANTs SyN transform (combining the affine and non‐linear warp components) to serve as an additional quantitative comparison of EPImix and corresponding single‐contrast acquisitions (henceforth referred to as log‐Jacobian).

### 2.4 Effects of resolution and spatial smoothness on the correspondence between EPImix and single‐contrast T_1_ ‐w scans

To evaluate the impact of spatial resolution on the correspondence between EPImix and single‐contrast T_1_‐w scans, as well as the test–retest reliability of EPImix contrasts and derived measures, we downsampled voxelwise data within regions of interest (ROIs). To investigate the impact of ROI size within the atlas used, we used both a high‐resolution multi‐modal parcellation (MMP) of cortex into 360 ROIs, constructed by Glasser et al. (2016), as well as its downsampled low‐resolution version into 44 larger regions. These two atlases are hereafter referred to as ‘MMP high‐resolution’ (or ‘MMP high‐res.’) and ‘MMP low‐resolution’ (or ‘MMP low‐res.’), respectively. For details, see Figure S2.

Due to the reduced FoV of EPImix scans, resulting in missing portions of the inferior temporal and/or superior parietal lobe in certain participants, we only included voxels present (i.e., non‐zero) in at least 80% of EPImix scans in voxelwise analyses (i.e., 76/95 participants). For regional analyses, we only included ROIs where at least 80% of voxels contained non‐zero values in at least 80% of participants. This resulted in analyses using 297/360 regions from the Glasser et al., 2016 atlas, and 32/44 regions from its downsampled version. For details, see Figure S3.

Regional values were generated by calculating the median values of unsmoothed voxel‐wise EPImix contrasts, single‐contrast T_1_‐w scans and log‐Jacobians within atlas masks registered to the same MNI space, excluding zero‐valued voxels. We subsequently performed analyses at the spatial resolution of voxels (both spatially smoothed and unsmoothed), 297 and 32 ROIs, as described below. Additionally, voxel‐wise analyses were performed and/or visualised using voxels within a mask defined by the MNI brain (dilated once), as well as cortical grey matter (GM) voxels (defined as voxels belonging to one of the regions of the cortical MMP atlases used).

Furthermore, to evaluate the impact of spatial smoothness on the correspondence between EPImix and corresponding single‐contrast scans, we smoothed voxelwise EPImix and single‐contrast T_1_‐w scans using three different Gaussian kernels—2, 4, and 6 mm full‐width at half‐maximum (FWHM; using Python *nilearn*; Abraham et al., 2014).

### 2.5 Correspondence between EPImix and single‐contrast scans

We quantified correspondence between matching EPImix and single‐contrast T_1_‐w scans in several ways. (All instances of correlation refer to Spearman's correlation coefficient ρ.).

To evaluate the extent of spatial correspondence between EPImix and single‐contrast scans, we correlated corresponding log‐Jacobians and T_1_‐w intensities at the voxel and ROI level, across subjects.

Further, to determine whether the correspondence between matching EPImix and single‐contrast T_1_‐w scans is higher within than between participants, we calculated measures of ‘differential identifiability’ (Amico & Goñi, 2018). This is defined as the median correlation of participants' scans from one modality to their own scan from the other modality (i.e., within‐participant correlation; $\rho_{\text{within}}$), minus the median correlation between modalities of non‐corresponding participants (i.e., between‐participant correlation; $\rho_{\text{between}}$):

(2) $$I_{\text{diff}}=\mathrm{Md}\left(\rho_{\text{within}}\right)-\mathrm{Md}\left(\rho_{\text{between}}\right)$$

We additionally defined an individual index of differential identifiability, as the fraction of times that between‐subject scan correlations are smaller than within‐subject scan correlations. We calculated this measure twice for each participant and spatial resolution, to quantify both the individual identifiability of a single‐contrast T_1_‐w scan relative to EPImix T_1_‐w scans, and of an EPImix T_1_‐w scan relative to single‐contrast T_1_‐w scans. This individual measure of identifiability is related to discriminability, as defined by Bridgeford et al. (2020). We note that while (individual) identifiability based on log‐Jacobians is desirable as these maps encode inter‐individual differences in brain size and shape, the interpretation of identifiability based on T_1_‐w scan intensity is more complex (for details, see Section 4).

When correlating values at the regional level, we used a spatial permutation test to construct realistic null models of spatial correspondence. Specifically, these null models test whether correspondence between two cortical maps might be driven by spatial autocorrelation and hemispheric symmetry of these maps (null hypothesis; H_0_), or whether there is inherent spatial correspondence over and above these potential confounds (alternative hypothesis; H_1_). For details regarding the generation of regional spatial permutations, see (Markello & Misic, 2020; Váša et al., 2018) and Supporting Information.

As a final comparison between contrasts, we used regional data from EPImix and single‐contrast log‐Jacobians as well as T_1_‐w intensities to construct structural covariance matrices, by cross‐correlating median regional values across subjects (Alexander‐Bloch et al., 2013; Evans, 2013). We quantified correspondence between the upper triangular parts of the structural covariance matrices using correlation, and visualised networks from both modalities using (thresholded) network diagrams. We further contextualised the correspondence between networks using a mapping of high‐resolution MMP atlas regions to intrinsic connectivity networks derived by Yeo et al., 2011, previously defined in Váša et al., 2020 (for details of the mapping, see Supporting Information).

### 2.6 EPImix MSNs

We further explored the possibility of constructing MSNs (Seidlitz et al., 2018) from EPImix, by correlating regional contrast values between pairs of regions within subjects. EPImix‐derived MSNs provide a proxy measure of connectivity, which could serve both to complement measures of regional anatomy in driving the adaptive imaging process, and as a rapid brain network estimate in other applications. We used seven maps per participant to construct EPImix‐derived MSNs, including six EPImix contrasts as well as the log‐Jacobian derived from transforming EPImix T_1_‐w scans to MNI space. Regional values were normalised within each participant and contrast using the number of absolute deviations around the median, a non‐parametric equivalent of the *Z*‐score (Leys, Ley, Klein, Bernard, & Licata, 2013); for a vector of regional values x:

(3) $$Z_{\text{non}-\mathrm{par}}=\frac{x-\mathrm{Md}\left(x\right)}{\mathrm{MAD}\left(x\right)}$$

where $\mathrm{Md}\left(\right)$ corresponds to the median, and $\mathrm{MAD}\left(\right)$ to the median absolute deviation. Finally, normalised regional values were correlated using Spearman's ρ across maps (contrasts), within participants, to create individual MSNs.

We compared EPImix‐derived MSNs to conventional MSNs, derived from FreeSurfer reconstructions of single‐contrast T_1_‐w scans. MSNs have previously been reconstructed from 10 morphometric features derived from high‐resolution multi‐modal MRI data (Seidlitz et al., 2018), as well as five features derived from single‐contrast T_1_‐w scans (King & Wood, 2020). We used the FreeSurfer *recon‐all* command to reconstruct cortical surfaces (Fischl, Sereno, & Dale, 1999), followed by visual quality control; one of the 66 participants with both EPImix and single‐contrast T_1_‐w scans available was excluded due to a failed surface reconstruction, resulting in the use of 65 participants for this analysis. Subsequently, seven FreeSurfer‐generated quantitative measures were extracted from each region of both the high‐resolution and low‐resolution MMP atlases used: surface area, GM volume, cortical thickness, mean curvature, Gaussian curvature, folding index, and curvature index. Each measure was normalised using the same non‐parametric approach as EPImix MSNs (Equation (3)), and individual MSNs constructed using Spearman's ρ across regional normalised measures. We then compared EPImix‐derived and standard MSNs using Spearman's ρ correlations—of group‐averaged MSNs (across all edges, and within and between intrinsic connectivity networks), as well as within individual participants.

Finally, to explore the value of EPImix MSNs, we quantified the variance in participant age and sex explained by MSN edges using linear regression, in the full sample of (95) participants with EPImix scans. The explained variance score was calculated within five‐fold age‐stratified cross‐validation, with a resulting median value (across folds) calculated for each MSN edge.

### 2.7 Test–retest reliability of EPImix scans

We quantified test–retest reliability of EPImix scans using 10 within‐session test–retest EPImix scans. We quantified test–retest reliability using the intraclass correlation coefficient (ICC); specifically, we used the one‐way random effects model for the consistency of single measurements, that is, ICC(3,1), hereafter referred to as ICC (Chen et al., 2018). We calculated the ICC using voxel‐wise data, ROI‐averaged data and MSN edges.

## 3 RESULTS

### 3.1 Evaluation of a rapid processing pipeline

We sequentially evaluated the impact of four processing steps on the speed and quality of registration, using the Mindboggle‐101 dataset (Klein & Tourville, 2012). At each step, we recorded the processing time and the quality of overlap (between our custom registrations of DKT atlas labels and manual labels released with the Mindboggle dataset) using the Dice coefficient. These two measures are intended to inform (rather than determine) the selection of processing steps (see also Section 4).

**FIGURE 3:**
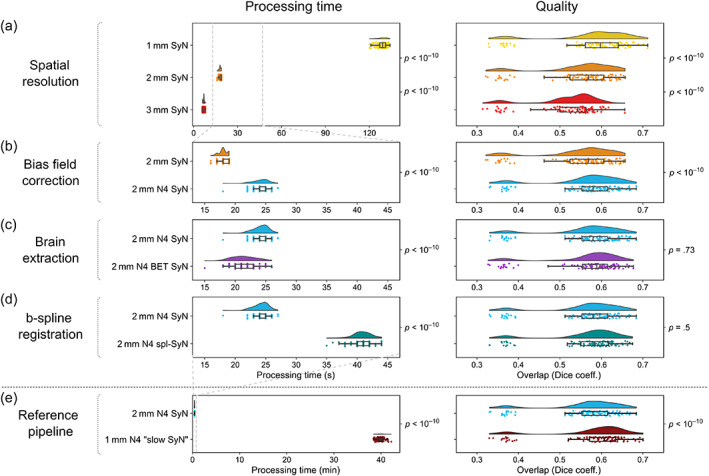
Evaluation of processing time and quality of registration using the Mindboggle dataset. The effect of four processing steps was evaluated sequentially; for each step, both processing time and quality were taken into account to select one of the options, before proceeding to the next step. *p*‐values adjacent to neighbouring raincloud plots correspond to the (paired) Wilcoxon signed‐rank test between corresponding data (testing whether evaluated methods differ significantly in processing time or registration quality [H_1_], or whether there is no statistical difference between these values [H_0_]). (a) Spatial resolution. (b) Bias field correction. (c) Brain extraction. (d) B‐spline SyN registration. (e) An additional reference pipeline was evaluated, to benchmark any reduction in quality resulting from optimising steps a–d for speed. *p*‐values were not corrected for multiple comparisons, due to the sequential nature of evaluated steps. We note that even stringent multiple comparisons correction has no qualitative impact on the results. For Bonferroni‐corrected *p*‐values, as well as median differences in both processing time and quality between pairs of compared pipelines, see Table S1

We first evaluated the impact of spatial resolution of the data. An isotropic resolution of 1 mm results in the most accurate registration, but is potentially too slow to be run in real‐time (processing time Md [Q_1_,Q_3_] = 129 [127,131] s). The processing of the images with 2 mm isotropic resolution is sufficiently fast (Md [Q_1_,Q_3_] = 18 [18,19] s) and was therefore chosen (Figure 3a). We next inspected the impact of bias field correction (on 2 mm isotropic resolution scans), using the ANTs N4 algorithm. We found that bias field correction improved registration quality at a relatively low time cost (Md [Q_1_,Q_3_] = 24 [24,25] s) and was therefore included as a processing step (Figure 3b). Subsequently, we explored the application of a brain extraction algorithm (to the 2 mm isotropic resolution scans following bias field correction) using FSL BET. Brain extraction results in a marginally faster registration (Md [Q_1_,Q_3_] = 21 [20,23] s), but with no gain in quality (Figure 3c). Combined with the fact that brain extraction might fail and need to be re‐run with alternative parameters, it was not included in our processing pipeline. Finally, we evaluated the use of ANTs b‐spline SyN registration (instead of the ‘standard’ ANTs SyN algorithm). This results in a noticeably slower registration, without a gain in quality (Md [Q_1_,Q_3_] = 41 [40,42] s); therefore, the standard ANTs algorithm was preferred (Figure 3d).

To benchmark the potential loss in quality resulting from the above selection of a fast processing pipeline, we evaluated an additional ‘reference’ pipeline, solely optimised for quality. This consisted of 1 mm scans, ANTs N4 bias field correction and registration using a slower (but more accurate) version of the ANTs SyN algorithm. As expected, this pipeline was far slower (Md runtime [Q_1_,Q_3_] = 39.9 [39.4,40.3] min), and only resulted in a marginal increase in registration quality (Figure 3e).

For each processing pipeline, we additionally calculated the Dice coefficient for individual regions of the DKT atlas. This showed a relatively spatially homogenous impact of processing steps on registration quality overall; for details, see Figure S4.

**FIGURE 4:**
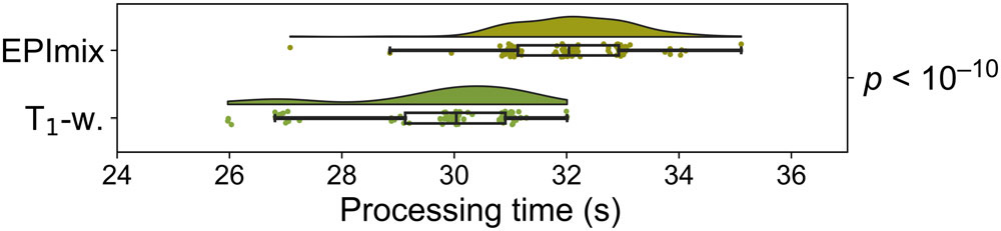
Processing time for EPImix and single‐contrast T_1_‐w scans. The *p*‐value corresponds to the (unpaired) Mann–Whitney *U* test (testing whether processing times differ for EPImix and single‐contrast T_1_‐w scans [H_1_], or whether there is no statistical difference between these values [H_0_]). Note that a small amount of jitter was added to data to better distinguish the distribution of integer‐valued data‐points

We next applied the selected processing pipeline, consisting of ANTs N4 bias field correction and ANTs SyN registration, to EPImix and corresponding single‐contrast T_1_‐w scans (the EPImix scans were not downsampled but registered to a 2 mm isotropic MNI template brain directly; the single‐contrast T_1_‐w scans were downsampled to 2 mm isotropic resolution prior to registration). Application of the selected processing pipeline to EPImix and single‐contrast T_1_‐w scans resulted in rapid processing of both acquisitions (EPImix processing time Md [Q_1_,Q_3_] = 32 [31,33] s, single‐contrast T_1_‐w processing time Md [Q_1_,Q_3_] = 30 [29,31] s; Figure 4).

### 3.2 Correspondence between EPImix and single‐contrast T_1_ ‐weighted scans

We evaluated correspondence between EPImix and single‐contrast scans using both log‐Jacobians extracted from transformations of T_1_‐w scans to MNI standard space, and T_1_‐w scan intensities. In the main text, we report results of log‐Jacobian comparisons as well as summary results for T_1_‐w intensities; full details for comparisons of T_1_‐w intensities are reported in Supporting Information.

We restricted analyses of EPImix and single‐contrast T_1_‐w scans to voxels with coverage in at least 80% participants (199′870/269′462 = 74.2% voxels in the MNI brain mask, and 64′370/78′247 = 82.3% voxels in the cortical GM mask), and regions where at least 80% voxels were non‐zero in at least 80% participants (297/360 = 82.5% regions in the high‐resolution MMP atlas, 32/44 = 72.7% regions in the low‐resolution MMP atlas). For details regarding participant overlap at voxels and regions, see Figure S3.

**FIGURE 5:**
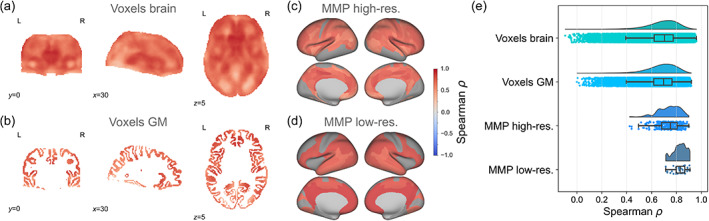
Local correspondence of log‐Jacobians across participants. Spearman's correlations between log‐Jacobians of rapidly‐processed T_1_‐w scans from the EPImix sequence and a single‐contrast acquisition, using data of 66 participants. Correlations are depicted: at the voxel level for (a) the whole brain, and (b) cortical grey matter, as well as within ROIs of (c) the high‐resolution and (d) the low‐resolution multi‐modal parcellation atlas. (e) Distributions of correlations at each spatial resolution considered (as depicted in panels a–d). (At the regional level, median regional values were extracted prior to calculation of correlations for each region.)

When evaluating correspondence between EPImix and single‐contrast T_1_‐w scans, we first calculated the correlation, across participants, of the log‐Jacobian value at each voxel or region. Most correlations were strong and positive, including Md(*ρ*) [Q_1_,Q_3_] = 0.70 [0.62,0.77] at the voxel level (0.70 [0.62,0.76] in the GM), 0.75 [0.68,0.81] at the level of regions of the high‐resolution MMP atlas, and 0.83 [0.8,0.87] for the low‐resolution atlas (Figure 5). Most or all correlations were statistically significant (*p*_FDR_ ≤.05 at >99% voxels for both brain voxels and GM voxels, and for all [100%] regions of both the high‐ and low‐resolution MMP atlases). Analogous comparisons using T_1_‐w intensities yielded lower correlations, including Md(*ρ*) [Q_1_,Q_3_] = 0.17 [0.05,0.29] within all brain voxels and 0.22 [0.11,0.32] within GM voxels, as well as 0.19 [0.13,0.26] within ROIs of the high‐resolution MMP atlas and 0.16 [0.12,0.19] for regions of the low‐resolution MMP atlas (Figure S5). Far fewer of these correlations were significant (*p*_FDR_ ≤.05 at 20% brain voxels and 31% GM voxels, and for only 3% regions of the high‐resolution MMP atlas and no [0%] regions of the low‐resolution MMP atlas).

**FIGURE 6:**
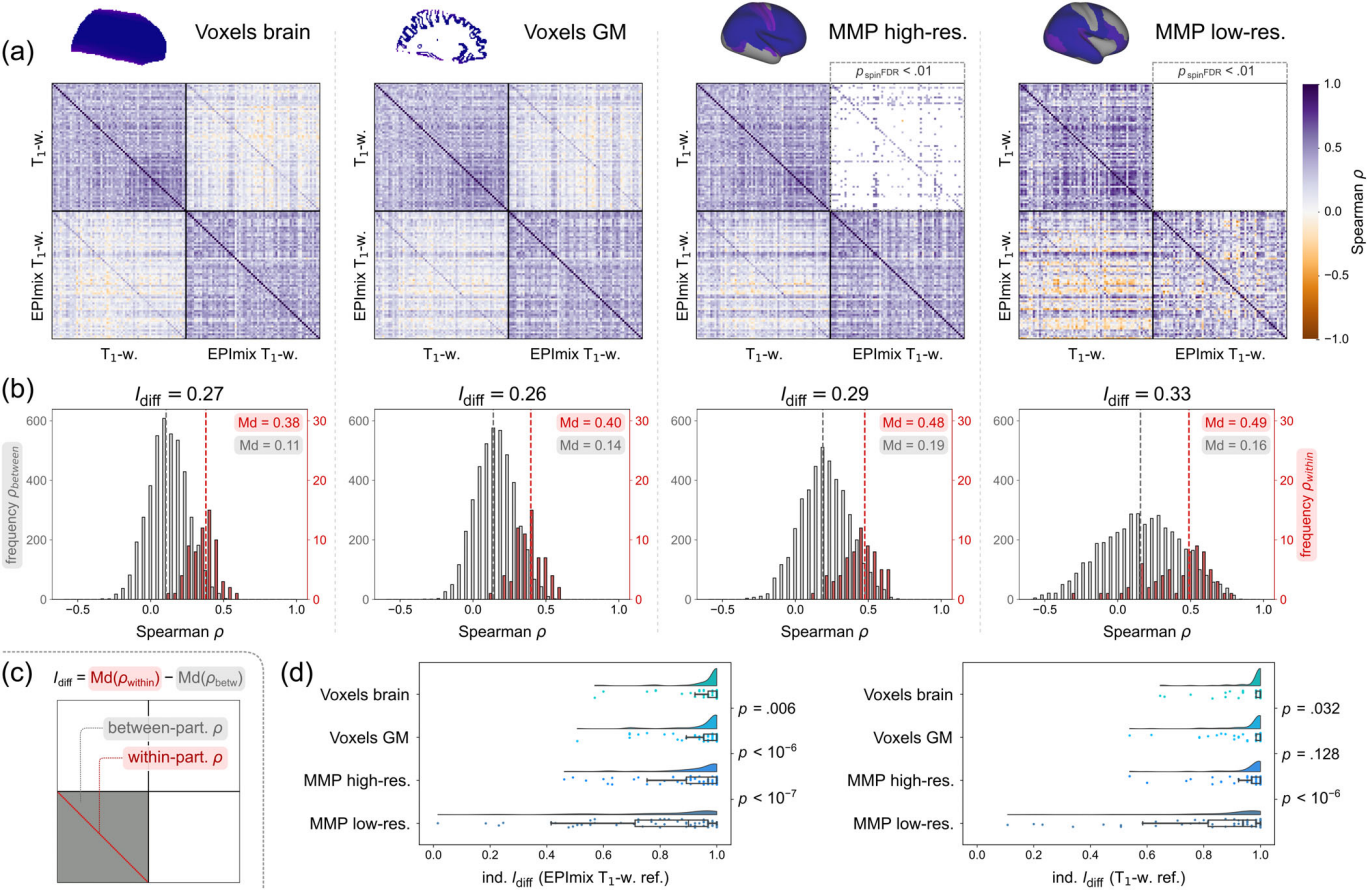
Participant identifiability across EPImix and single‐contrast scans, using log‐Jacobians. Between‐participant correlations and identifiability were investigated using four types of data, at three spatial resolutions (columns in a,b, rows in d): all brain voxels, cortical grey matter voxels, regions of the high‐resolution multi‐modal parcellation (MMP) atlas, and regions of the low‐resolution MMP atlas. (a) Spearman's correlations between EPImix and single‐contrast log‐Jacobians, within and between participants. Cross‐contrast correlations at the level of ROIs were benchmarked using a null model controlling for contiguity and spatial autocorrelation (upper triangular blocks). (b) Differential identifiability of contrasts, defined as the difference between the median within‐participant correlation (right/red *y*‐axes) and the median between‐participant correlation (left/grey *y*‐axes), as illustrated in c). (d) Individual identifiability, defined as the fraction of times that the within‐participant correlation is higher than between‐participant correlations, either identifying the log‐Jacobian of a single‐contrast T_1_‐w scan relative to log‐Jacobians of EPImix T_1_‐w scans (EPImix T_1_‐w ref.), or vice‐versa (T_1_‐w ref.). *p*‐values correspond to the (paired) Wilcoxon signed‐rank test between neighbouring distributions (testing whether different spatial resolutions of data lead to differences in individual identifiability (H_1_), or whether there is no statistical difference between these values (H_0_))

We next quantified the within‐ and between‐participant correspondence of EPImix and single‐contrast log‐Jacobians (Figure 6a). We calculated global identifiability, as the difference of the median between‐participant correlation and median within‐participant correlation (Figure 6b; relevant parts of the correlation matrices are depicted in Figure 6c). Differential identifiability was similar across types of data used, with highest identifiability at the level of low‐resolution regions (*I*_diff_ = 0.49–0.16 = 0.33), closely followed by high‐resolution regions (*I*_diff_ = 0.48–0.19 = 0.29), brain voxels (*I*_diff_ = 0.38–0.11 = 0.27), and finally GM voxels (*I*_diff_ = 0.40–0.14 = 0.26) (Figure 6b). For regional data, we additionally used a null model relying on spherical ‘spin’ permutation of cortical regions to account for spatial autocorrelation of the data when quantifying spatial correspondence between contrasts. Within the high‐resolution atlas, 52/66 = 78.8% of within‐participant correlations survived the FDR‐corrected permutation test, compared to 406/4290 = 9.5% of between‐participant correlations. Within the low‐resolution atlas, no within‐ or between‐participant correlations survived this thresholding procedure (Figure 6a). Finally, we calculated individual‐level identifiability, as the fraction of times that within‐participant scan correlations are higher than between‐participant scan correlations, using one of the contrasts as a reference (Figure 6d). For example, identifiability of an individual EPImix T_1_‐w scan is maximal (=1) when the correlation between that scan and the same participants' single‐contrast T_1_‐w scan is higher than all correlations to other participants' single‐contrast T_1_‐w scans. Individual identifiability was highly similar when using EPImix T_1_‐w scans and single‐contrast T_1_‐w scans as reference. In contrast with global differential identifiability, individual participants were most identifiable at the level of brain voxels, with high individual identifiability at the level of GM voxels and high‐resolution ROIs as well; low‐resolution regions had comparatively lower individual identifiability (Figure 6d). Analogous analyses using T_1_‐w scan intensities yielded highest differential and individual identifiability at the level of GM voxels (*I*_diff_ = 0.24; Md[ind. *I*_diff_] = 1), with lower correspondence at other spatial resolutions; for details, see Figure S6.

We additionally inspected the effect of voxel‐wise smoothing of T_1_‐w intensity data, using 2, 4, and 6 mm FWHM kernels. The effect of smoothing was to reduce voxel‐wise correspondence across subjects (i.e., the analogue of Figure 5a,b), and to reduce differential identifiability due to a greater increase in the magnitude of between‐participant correlations than within‐participant correlations; for details, see Figure S7.

For a summary of median within‐ and between‐participant correspondence, and global and individual identifiability across spatial resolutions and types of data used (log‐Jacobians and T_1_‐w intensities), see Table 3.

**TABLE 3:** Identifiability of log‐Jacobians and T_1_‐w intensities at different spatial resolutions

		Md(*ρ*_with_))	Md((*ρ*_betw_))	*I*_diff_	Ind. *I*_diff_ T_1_‐w ref. Md [Q_1_,Q_3_]	Ind. *I*_diff_ Em ref. Md [Q_1_,Q_3_]
Log‐Jacobian	Voxels brain	0.38	0.11	0.27	1.0 [0.98,1.0]	1.0 [0.97,1.0]
	Voxels GM	0.40	0.16	0.24	1.0 [0.98,1.0]	1.0 [0.95,1.0]
	MMP high‐res.	0.48	0.19	0.29	1.0 [0.97,1.0]	0.97 [0.89,1.0]
	MMP low‐res.	0.49	0.16	0.33	0.94 [0.82,0.98]	0.90 [0.71,0.97]
T_1_‐w intensity	Voxels brain	0.62	0.50	0.12	1.0 [1.0,1.0]	1.0 [1.0,1.0]
	Voxels GM	0.47	0.23	0.24	1.0 [1.0,1.0]	1.0 [1.0,1.0]
	MMP high‐res.	0.61	0.43	0.19	1.0 [1.0,1.0]	1.0 [1.0,1.0]
	MMP low‐res.	0.78	0.66	0.12	0.91 [0.79,0.98]	0.88 [0.72,0.97]
T_1_‐w GM smoothed	2 mm FWHM	0.50	0.26	0.24	1.0 [1.0,1.0]	1.0 [1.0,1.0]
	4 mm FWHM	0.59	0.38	0.21	1.0 [1.0,1.0]	1.0 [1.0,1.0]
	6 mm FWHM	0.64	0.49	0.16	1.0 [1.0,1.0]	1.0 [1.0,1.0]

*Note*: From left to right, columns correspond to: median within‐participant correlation, median between‐participant correlation, global identifiability, individual identifiability with T_1_‐w scans as reference, and individual identifiability with EPImix T_1_‐w scans as reference. From top to bottom, blocks correspond to log‐Jacobians and T_1_‐w intensities (with rows corresponding to spatial resolution), as well as the effect of smoothing voxel‐wise GM T_1_‐w intensity maps (with rows corresponding to the width of the smoothing kernel).

### 3.3 Correspondence of structural covariance networks across contrasts

We next inspected the correspondence between structural covariance networks, constructed by correlating log‐Jacobians (or T_1_‐w intensities) between all pairs of regions, across participants.

**FIGURE 7:**
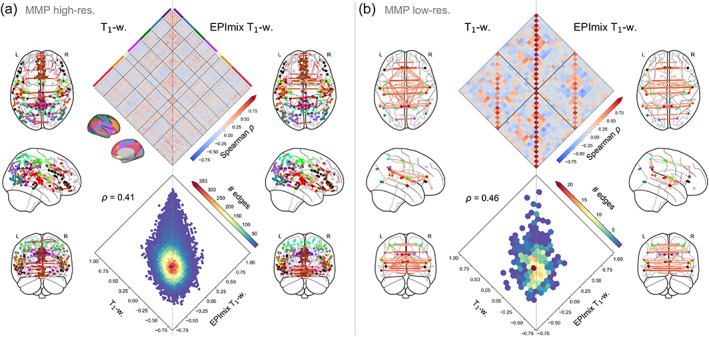
Structural covariance networks constructed from EPImix and single‐contrast log‐Jacobians. (a) Structural covariance networks constructed using the high‐resolution MMP atlas (297 regions). The diamond plot (top) is ordered according to regional membership of the seven canonical intrinsic connectivity networks derived by Yeo et al. (2011). Network diagrams depict the strongest 0.3% correlations. (b) Structural covariance networks constructed using the low‐resolution MMP atlas (32 regions). Network diagrams depict the strongest 10% correlations. For a comparison of high‐resolution structural covariance within intrinsic connectivity networks, see Figure S10

Structural covariance networks constructed using log‐Jacobians exhibited similar hallmarks of organisation to structural covariance networks commonly constructed from regional cortical thickness or GM volume data, such as strong long‐range inter‐hemispheric correlations between homotopic regions (Figure 7). The upper triangular parts of these matrices exhibited moderate correspondence between acquisitions, both for the high‐resolution atlas (Spearman's *ρ* = 0.41), and the low‐resolution atlas (Spearman's *ρ* = 0.46). Correspondence varied across intrinsic connectivity networks (Md(*ρ*) [Q_1_,Q_3_] = 0.41 [0.28, 0.49]), with highest correlations within visual, somatomotor and limbic networks (Figure S10). Conversely, structural covariance networks constructed using T_1_‐w scan intensities showed lower correspondence between acquisitions, particularly within the high‐resolution atlas; this was likely due to unusually high short‐range correlations clustered in the frontal cortex in the EPImix T_1_‐w data (Figures S8 and S10).

### 3.4 EPImix MSNs

**FIGURE 8:**
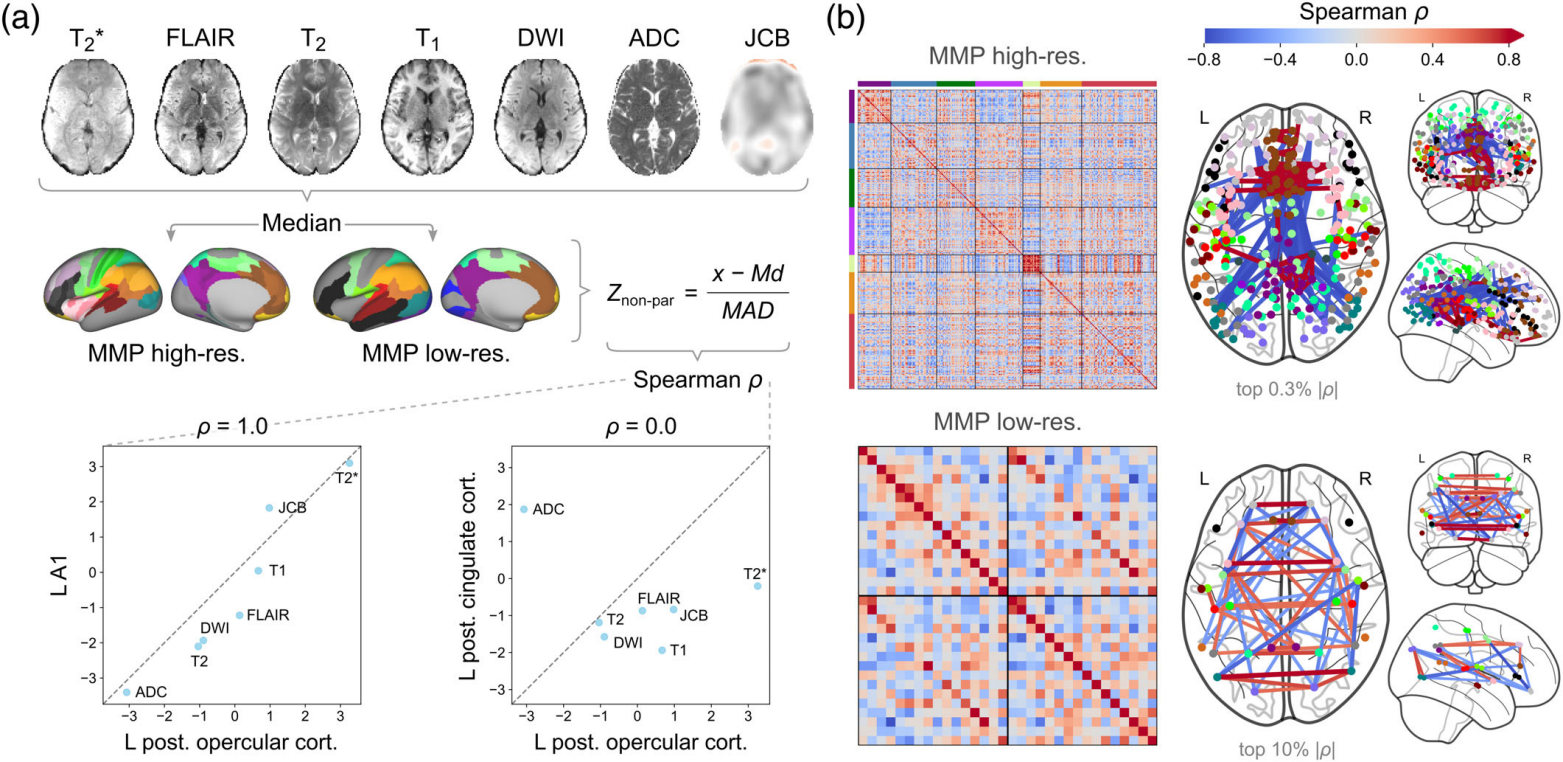
Morphometric similarity networks (MSNs) constructed from EPImix contrasts and log‐Jacobians. (a) MSN construction. Seven maps, including six EPImix contrasts and a log‐Jacobian map (obtained from the warp of the T_1_‐w contrast to MNI space) were used for network construction. Median values of each map within each region of a high‐resolution and a low‐resolution atlas were calculated, before normalisation (within participants, across regions) using a non‐parametric equivalent of the *Z*‐score (MAD, median absolute deviation; Md, median). Two example correlations from the low‐resolution atlas are shown: high morphometric similarity of left posterior opercular cortex to left early auditory cortex (A1), and low morphometric similarity to the left posterior cingulate cortex. b) Average MSNs (across participants), constructed using the high‐resolution atlas (top; strongest 0.3% absolute correlations shown) and low‐resolution atlas (bottom; strongest 10% absolute correlations shown). For comparisons of MSNs derived from EPImix contrasts to conventional MSNs derived from FreeSurfer reconstructions of T_1_‐w scans, see Figures S9 and S10

As a unique application of the multicontrast EPImix sequence, we explored the possibility of constructing MSNs (Seidlitz et al., 2018). We constructed individual MSNs by correlating nonparametrically normalised regional values of the six EPImix contrasts as well as the log‐Jacobian (seven regional features in total), between all pairs of regions. For example, in the low‐resolution MMP atlas, the left posterior opercular cortex showed high morphometric similarity to the left early auditory cortex (A1), but low similarity to the left posterior cingulate cortex (Figure 8a). MSNs showed high positive correlations between homotopic pairs of regions (Figure 8b).

We further compared EPImix‐derived MSNs to standard MSNs obtained from FreeSurfer reconstructions of single‐contrast T_1_‐w scans, in 65 participants. The group‐average MSNs showed weak correspondence, both for the high‐resolution atlas (Spearman's ρ = 0.21) and for the low‐resolution atlas (Spearman's ρ = 0.26). Correspondence of edges within and between intrinsic connectivity networks was variable; Md(*ρ*) [Q_1_,Q_3_] = 0.17 [0.12, 0.22]. Correspondence within individual participants covered a range of (low) values, for both atlases; high‐resolution MMP Md(*ρ*) [Q_1_,Q_3_] = 0.085 [0.074, 0.10], low‐resolution MMP Md(*ρ*) [Q_1_,Q_3_] = 0.12 [0.076, 0.16]. For details, see Figures S9 and S10B.

Finally, we explored the relationships of MSN edges to age and sex using all 95 participants with EPImix scans. At each edge of both the high‐resolution and low‐resolution MSN networks, we predicted edge strength (correlation) as a function of age and sex, using age‐stratified five‐fold cross‐validation. We fitted each model to 80% (76/95) participants, and used the remaining 20% (19/95) participants to extract an explained variance score. Edge‐wise median explained variance reached maximal values of 0.35 for high‐resolution MSN edges, and 0.18 for low‐resolution MSN edges.

### 3.5 EPImix test–retest reliability

**FIGURE 9:**
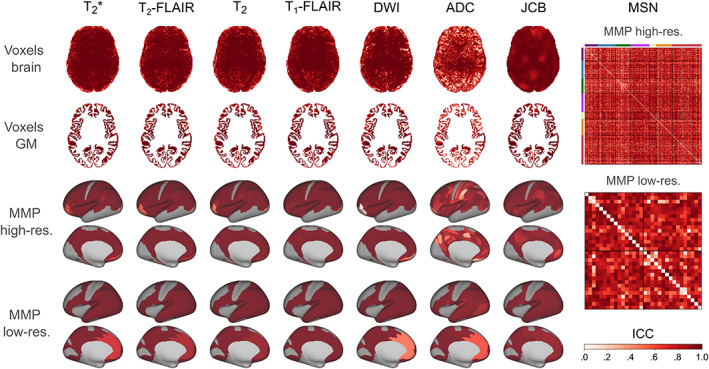
Test–retest reliability of rapidly‐processed EPImix scans. Reliability was assessed using 10 within‐session test–retest scans, for the six EPImix contrasts and the log‐Jacobian (JCB), at the level of voxels, and high‐ and low‐resolution MMP atlases, as well as for morphometric similarity networks (MSNs) at both atlas resolutions. Reliability was quantified using the one‐way random effects model for the consistency of single measurements, that is, ICC(3,1). (Note that the EPImix T_1_‐FLAIR contrast is referred to as the EPImix T_1_‐w contrast/scan throughout the text.)

The final analysis of the study consisted in quantifying the test–retest reliability of rapidly processed EPImix contrasts, log‐Jacobians and MSNs, using within‐session test–retest data from 10 participants. Reliability was consistent across levels of spatial resolution, including voxels and ROIs, and generally very high (Figure 9 and Table 4). Among contrast maps, reliability was lowest for the ADC; all other maps showed high reliability. MSN edges showed high reliability, with a few exceptions. For median and quartile ICC values, see Table 4.

**TABLE 4:** Test–retest reliability of values derived from 10 EPImix scans

ICC: Md [Q_1_,Q_3_]	Voxels brain	Voxels GM	MMP high‐res.	MMP low‐res.
T_2_*	0.97 [0.93,0.99]	0.97 [0.93,0.99]	1.0 [0.99,1.0]	1.0 [1.0,1.0]
T_2_‐FLAIR	0.97 [0.91,0.99]	0.96 [0.89,0.98]	1.0 [0.99,1.0]	1.0 [1.0,1.0]
T_2_	0.96 [0.88,0.99]	0.94 [0.86,0.98]	0.99 [0.98,1.0]	1.0 [0.99,1.0]
T_1_‐FLAIR	0.96 [0.9,0.99]	0.94 [0.88,0.97]	0.99 [0.98,1.0]	1.0 [0.99,1.0]
DWI	0.96 [0.86,0.99]	0.94 [0.80,0.98]	0.99 [0.98,1.0]	1.0 [0.99,1.0]
ADC	0.78 [0.55,0.91]	0.74 [0.48,0.89]	0.91 [0.79,0.96]	0.97 [0.93,0.99]
log‐Jacobian	0.96 [0.92,0.98]	0.96 [0.92,0.98]	0.97 [0.94,0.98]	0.98 [0.97,0.99]
MSN	N/A	N/A	0.81 [0.66,0.89]	0.83 [0.69,0.90]

*Note*: Reliability was assessed for six EPImix contrasts as well as the log‐Jacobian and MSNs, as Median [Q_1_,Q_3_] ICC(3,1). (Note that the EPImix T_1_‐FLAIR contrast is referred to as the EPImix T_1_‐w contrast/scan throughout the text.)

## 4 DISCUSSION

### 4.1 Rapid processing of MRI data

Using manually labelled scans from the Mindboggle dataset (Klein & Tourville, 2012), we first evaluated the impact of several processing steps on the processing time and quality of registration. The results informed our choice of ‘minimal’ pre‐processing pipeline, which included N4 bias field correction and ANTs SyN registration of non‐skull‐stripped scans at 2 mm isotropic resolution. This combination of processing steps resulted in very fast processing (<1 min on a typical computer) of both EPImix and single‐contrast T_1_‐w scans. Notably, the quality of registration achieved by this fast processing pipeline was only marginally lower than a far slower (~40‐min) reference pipeline optimised for quality. In addition to the relevance of these tools to novel acquisition paradigms such as adaptive multimodal imaging (Cole et al., 2019), our systematic evaluation of rapid processing steps should be informative for widespread studies of real‐time functional MRI such as neurofeedback (Watanabe et al., 2017) and neuroadaptive Bayesian optimisation (Lorenz et al., 2017), where analysis pipelines rely on rapid processing of structural scans.

We further demonstrated that EPImix scans processed using our rapid pipeline showed high test–retest reliability. It would be interesting, in future, to repeat these analyses using test–retest data acquired during different sessions and/or at different scanner sites, for a more stringent test of the reliability and robustness of our rapid processing approaches (Chen et al., 2018).

We note that we deliberately avoided combining the processing time and quality into a single metric, which would be used to determine the selection of an option at each processing step. This would require placing a numerical weight on the relative importance of each criterion; for example, ‘time is [half/twice/three times…] as important as quality’, which is not trivial, or indeed necessary. Consider the example of bias field correction, as evaluated here: Is the median gain of 0.011 in output quality (as measured by the Dice coefficient) worth an added median 6 s of processing time? In this case, we decided that it is—but crucially, we consider that this is not a decision that it is easy to automate. Moreover, as discussed at the start of the Section 2, there are other non‐quantifiable factors that may affect the design of a processing pipeline, such as the necessity for (manual) quality control and subsequent re‐running of processing steps. This would also be more likely for some steps (e.g., brain extraction, registration) than others (downsampling, bias field correction). Taken together, we hope that our quantitative evaluation may serve as a framework to guide the identification of desired processing steps, to design a customised fast processing pipeline on a case‐by‐case basis.

We also did not systematically evaluate all combinations of processing steps, in part due to the assumption that variation in registration quality (one of the underlying objective functions) will be relatively smooth along axes corresponding to each processing step (Lancaster et al., 2018); in other words, the interactions of the processing steps were assumed to be largely linear. Moreover, our evaluation was limited to a single algorithm per processing option. Use of other algorithms or software suites may have resulted in processing pipelines that run faster and/or result in higher quality registrations (Klein et al., 2009). In future, the multiverse of processing steps and algorithms could be more systematically and efficiently explored using adaptive methods, to simultaneously quantify the performance of different combinations of processing tools and identify optimal approaches (Dafflon et al., 2020).

Deep learning tools are another promising avenue for further optimisation of image processing. Such tools can substantially reduce the runtime of intensive processing steps (e.g., Henschel et al., 2020). Many of these tools require computationally expensive training on large datasets or are limited to specific applications, although new methods are being developed that are relevant to a broad range of tasks (Isensee et al., 2020). Another recent development is deep‐learning methods trained on synthetic data, which promise to generate accurate segmentation (Billot et al., 2020) and registration (Hoffmann et al., 2020) in seconds, without the usual requirements of large empirical training datasets. The runtime/quality trade‐off of these tools could be benchmarked using the tools presented here.

### 4.2 Correspondence between EPImix and single‐contrast scans

We quantified the correspondence between log‐Jacobians and T_1_‐w intensities derived from EPImix and single‐contrast scans. Correspondence was generally high, across different spatial resolutions (including voxels and ROIs) and higher within participants than between them, leading to high levels of participant identifiability. For log‐Jacobians, global identifiability was highest for regions of the low‐resolution MMP atlas, closely followed by other data resolutions; however, individual identifiability was similarly high at voxels and high‐resolution regions, but lower for low‐resolution regions. For T_1_‐w intensities, both global and individual identifiability were highest using unsmoothed GM voxels.

We note that the interpretation and desirability of (individual) identifiability differs between its application to log‐Jacobians and T_1_‐w intensities. Jacobian maps (of the nonlinear transformation of individual scans to standard MNI space) encode inter‐individual differences in brain size and shape, and hence high identifiability based on these maps is desirable. The interpretation of identifiability based on T_1_‐w scan intensity is more complex, as the aim of registration is to align tissue intensities between the participant's scan and the template (Toga & Thompson, 2001). Here, identifiability values derived from log‐Jacobians and T_1_‐w intensities tended to be inversely related, which aligns with the interpretation that T_1_‐w contrast identifiability is undesirable, while Jacobian identifiability is. We also note that raw scan intensities might be sensitive to noise, including known variability due to factors such as scanner site or scan parameters (Shinohara et al., 2014). Methods have been proposed to harmonise intensities across scans, which involve matching distribution intensities within a reference section of tissue and accordingly adjusting intensities in other tissue classes (Fortin et al., 2016; Shinohara et al., 2014). However, such methods assume that variability within the reference tissue class is undesirable and thus preclude hypothesis testing in this tissue. Taken together, our results suggest that the use of log‐Jacobians is preferred for quantitative analyses such as individual identifiability. High(est) identifiability at the level of ROIs indicates that this may be the optimal level of spatial resolution for further analyses of similar data. The specific choice of atlas to be used, including the spatial resolution of ROIs, will depend on specific questions—including factors such as the expected spatial extent of potential abnormalities in clinical studies as well as computational cost of analyses, which may grow with the number of ROIs considered.

As an additional means of comparing EPImix and single‐contrast scans, we constructed structural covariance networks from regional log‐Jacobians and T_1_‐w intensities, to inspect the feasibility of constructing structural covariance networks using rapidly processed (EPImix) data. In the absence of ground truth, we use high‐resolution T_1_‐w scans from the same participants and scanner as the ‘gold standard’ to derive these networks, and therefore assume that differences in structural covariance network organisation are due to the EPImix sequence missing some of the signal. A particularly useful application of these networks is the calculation of individual contributions to group structural covariance, as a single‐contrast network biomarker of disease (Nadig et al., 2021; Saggar et al., 2015). However, further work is required to disentangle differences between structural covariance networks derived from EPImix and single‐contrast T_1_‐w scans, to apply individual deviation methods to such networks and to establish the potential practical relevance of such approaches. In particular, given the modest between‐contrast correspondence and apparently abnormal organisation of structural covariance networks derived from T_1_‐w scan intensities, structural covariance networks constructed from regional log‐Jacobian values are a more promising avenue for further work.

An alternative approach for deriving measures of brain connectivity from EPImix scans relies on MSNs (Seidlitz et al., 2018), constructed from within‐participant correlations between regional morphometric features. Here we used six EPImix contrasts as well as the log‐Jacobian to derive individual MSNs. EPImix‐derived MSNs showed relatively weak correspondence to standard MSNs obtained from FreeSurfer reconstructions of single‐contrast T_1_‐w scans (King & Wood, 2020). This could be due to several factors, including a more informative nature of the quantitative measures of brain morphology derived from FreeSurfer (such as cortical thickness or GM volume) compared to EPImix contrast intensities, more accurate delineation of ROI boundaries by FreeSurfer and a lower resolution of EPImix compared to single‐contrast scans. Nevertheless, our rapidly‐derived MSNs showcase the possibility of constructing individual multicontrast brain networks within minutes of participants entering the scanner. Rapidly‐derived network estimates could potentially complement other measures in informing adaptive imaging paradigms, or serve as stand‐alone screening tests for diseases affecting brain connectivity. However, further work will be required to first disentangle factors that affect the (currently limited) correspondence between EPImix‐derived and conventional MSNs, and more importantly, to ascertain the practical value of both network types in a predictive (clinical) setting.

We limited our comparison to EPImix and single‐contrast T_1_‐w scans (and corresponding log‐Jacobians), due to lack of availability of high‐resolution single‐contrast data analogous to other EPImix contrasts for the same participants. Further work on developing rapid processing pipelines for additional sequences would be valuable, along with quantitative evaluations of within‐participant correspondence between other EPImix contrasts and corresponding single‐contrast scans. The methods used here to compare T_1_‐w scans within participants can easily be translated to these other sequences, or further used to compare quantitative measures derived from other rapidly acquired scans. This includes other rapid multicontrast sequences (Polak et al., 2020) as well as contrasts acquired on the recently developed low‐field Hyperfine scanner (Sheth et al., 2020), both of which could be compared to their high‐resolution counterparts using the tools described herein.

We note that an inherent limitation of data from the EPImix sequence (Skare et al., 2018) is a reduced quality compared to analogous single‐contrast scans; for example, the EPImix acquisition is fast in part due to its reduced matrix size. However, such sequences are not intended to replace high‐resolution data; instead, they might serve as rapid ‘screening‐tests’, or for further planning of conventional MRI acquisition (Skare et al., 2018) ‐ including in an adaptive acquisition paradigm (Cole et al., 2019).

### 4.3 Towards adaptive imaging

The processing pipeline explored here is fast enough to be used while participants are still in the scanner, satisfying one of the key conditions for practical implementation of adaptive imaging (Cole et al., 2019). For the specific development of adaptive multimodal imaging, another requirement is a criterion for selecting which imaging modality or contrast to acquire next, based on hitherto acquired rapidly processed data.

One such criterion can be the selection of modalities predicted to show greatest deviations relative to large normative datasets (Marquand et al., 2019), based on previously acquired scans as well as knowledge of covariance across modalities. Thus, it would be valuable to use large multimodal normative datasets, such as CamCAN (Taylor et al., 2017), Human Connectome Project (Van Essen et al., 2012) or UK Biobank (Miller et al., 2016), to investigate both normative deviations of rapidly processed data across modalities as well as covariance between modalities across participants. In this context, repetition of analyses across spatial scales, such as voxels and ROIs, could generate insights into the potential extent (and therefore nature) of abnormalities.

As a proof‐of‐concept of adaptive acquisition, we propose to use rapid processing and analysis of EPImix scans to determine which of the six contrasts show the greatest deviations from a normative population (Marquand et al., 2019). Subsequently, and while the participant is still in the scanner, these contrasts would be re‐acquired at higher resolution using single‐contrast sequences, in the order automatically determined by our rapid analysis algorithm. Subsequent ‘off‐line’ analyses could confirm the accuracy of the order of contrasts by extent of deviation from the norm, previously determined in real‐time.

Our suggested proof‐of‐concept application of EPImix (or similar rapidly‐acquired data) for adaptive imaging focuses on the selection of MRI contrasts or modalities, in line with initial proposals by Cole et al. (2019). However, adaptive imaging could also be applied within individual sequences, to optimise scan factors such as EPI readout, acceleration or inversion time, and adjust protocols to different populations to achieve optimal image quality.

Practical implementation of adaptive multimodal imaging would enable personalised neuroimaging examinations of patients. This would potentially lead to reduced scanning time and cost and consequently greater patient comfort, as well as to a decreased likelihood of recalling patients for further examinations (Cole et al., 2019). An added benefit of adaptive methods in the research context is the reduced likelihood of questionable practices such as P‐hacking or SHARKing (selecting hypotheses after results are known; Poldrack et al., 2017), due to the combination of data acquisition and analysis in a closed loop (Lorenz et al., 2017).

## 5 CONCLUSION

In summary, we explored the impact of several rapid processing steps on the runtime and quality of registration, and used results to inform the choice of steps forming a minimal processing pipeline. Subsequently, we quantified the correspondence between rapidly processed multicontrast EPImix and single‐contrast T_1_‐w scans, demonstrating that substantial quantitative information can be reliably extracted from the EPImix sequence in minutes. Finally, we explored the use of EPImix for the rapid construction of MSNs. Our work constitutes a step towards adaptive multimodal imaging, where real‐time scan processing and analysis can inform tailoring of neuroimaging examinations to individual patients.

## Supporting information


**Appendix** S1: Supporting information

## Data Availability

All processing and analysis code is available on FV's github, at https://github.com/frantisekvasa/epimix_rapid_processing. Mindboggle data is publicly available at https://osf.io/ydyxu/ (Klein & Tourville, 2012). Processed EPImix and single‐contrast T_1_‐w data, including contrast intensities and log‐Jacobians at voxels of the MNI brain, is available at https://doi.org/10.6084/m9.figshare.13862729 (Váša, 2021).
