# Supplementary material for: Rapid processing and quantitative evaluation of structural brain scans for adaptive multimodal imaging
Source: Hum Brain Mapp. 2021 Dec 24;43(5):1749–65. doi: 10.1002/hbm.25755 (PMC8886661; doi:10.1002/hbm.25755)
Supplement: Supplementary file 1 — Appendix S1: Supporting information [file HBM-43-1749-s001.pdf]

Supplementary Information for: Rapid processing and quantitative evaluation of structural brain scans for adaptive multimodal imaging

František Váša*, Harriet Hobday, Ryan A. Stanyard, Richard E. Daws, Vincent Giampietro, Owen O'Daly, David J. Lythgoe, Jakob Seidlitz, Stefan Skare, Steven C. R. Williams, Andre F. Marquand, Robert Leech[1], James H. Cole[1]

Supplementary Methods

Acquisition parameters

EPImix scans were collected from 95 participants (48 female, 47 male; age median [1st, 3rd Quartile] (Md [Q_1,Q_3]) = 25 [22,29] years; Supplementary Information (SI) Fig. S1), consisting of six contrasts (T_2*, T_2-FLAIR, T_2, T_1-FLAIR, DWI, ADC) acquired with the following parameters: T_2*: TE = 28.5 ms, TR = 2430 ms; T_2-FLAIR: TE = 113 ms, TR = 5797 ms, TI = 2751 ms; T_2, DWI & ADC: TE = 90.5 ms, TR = 2272 ms; T_1-FLAIR: TE = 16.5 ms, TR = 1300 ms, TI = 582 ms; flip angle = 90°, matrix size = 180 x 180, FoV = 240 mm, 32 slices, slice thickness = 3 mm, voxel resolution = 0.975 x 0.975 x 3 mm. The EPImix sequence includes an on-scanner motion correction step; the motion corrected images were used for further analyses. For further details regarding the EPImix sequence, see Skare et al. (2018). Additionally, for 10 participants, a second EPImix scan was acquired during the same session to investigate test-retest reliability.

We note that the EPImix acquisition is currently fixed to a 180 x 180 in-plane matrix size, so options for modifying the resolution during acquisition are limited. However, processing of EPImix scans with 0.9375 x 0.9375 x 3 mm resolution is sufficiently fast to be carried out without further downsampling.

Of the participants scanned with the EPImix sequence, 66 were additionally scanned, within the same session, with an IR-FSPGR T_1-weighted sequence (33 female, 33 male; age Md [Q_1,Q_3] = 25 [23,29.75] years; SI Fig. S1). Of these, 12 were scans with the following parameters: TE = 3.172 ms, TR = 8.148 ms, TI = 450 ms, flip angle = 12°, matrix size = 256 x 256, FoV = 256 mm, 164 slices, slice thickness = 1 mm, voxel resolution 1 x 1 x 1 mm; and 54 were scans with the following parameters: TE = 3.016 ms, TR = 7.312 ms, TI = 400 ms, flip angle = 11°, matrix size = 256 x 256, FoV = 270 mm, 196 slices, slice thickness = 1.2 mm, voxel resolution = 1.05 x 1.05 x 1.2 mm.

We note that the EPImix sequence includes a T_1-FLAIR contrast, while the high-resolution single-contrast scan is an IR-FSPGR sequence. In both sequences, the signal from CSF is low or nulled - in the case of T_1-FLAIR, an inversion recovery sequence is used in which the inversion time is chosen such that the magnetisation from CSF passes through zero. In the IR-FSPGR T1-weighted sequence, CSF also has low signal. In this case, however, this is not due to choice of inversion time, but due to the relationship between contrast, flip angle and repetition time. Importantly, we refer to both contrasts as simply "T_1-weighted", or "T_1-w".

Spherical permutations of ROIs

Spherical permutations were generated by randomly rotating a projection of ROI centroids on the (FreeSurfer) sphere, before mapping rotated ROIs to the nearest unrotated ones. Mirrored rotations were applied to the contralateral hemisphere, resulting in a permutation which controls for spatial autocorrelation and hemispheric symmetry of regions (Váša et al., 2018; Alexander-Bloch et al., 2018; Markello and Misic, 2020). P-values for the correlation between two regional maps were obtained by comparing the empirical value of Spearman's ρ to a null distribution of Spearman correlations, generated by correlating one of the empirical maps to a set of 10,000 spatially permuted versions of the other map; these "spin-test" P-values are referred to as P_{spin}. Spin-test P-values were additionally corrected for multiple comparisons using the false discovery rate (FDR; Benjamini and Hochberg, 1995).

Intrinsic connectivity networks

We used a mapping of 7 intrinsic connectivity networks derived by Yeo et al. (2011) to the high-resolution MMP atlas, to contextualise our results. This mapping, previously described and used in Váša et al. (2020), was obtained as follows. We first computed surface overlap (at the vertex level, using CIVET software; Ad-Dab'bagh et al., 2006) between each MMP atlas parcel and each intrinsic connectivity network, before assigning each MMP atlas parcel to the network that it overlapped most. Parcels of the high-resolution MMP atlas excluded from analyses due to limited FoV in EPImix scans (Fig. S3) were also excluded from intrinsic connectivity network visualisations and analyses.

*Corresponding author
Email address: fdv247@gmail.com (František Váša)
[1]These authors have contributed equally.

Correspondence between EPImix and single-contrast T_1-weighted scan intensities

Local correlations of T_1-w intensities were generally positive. At the voxel level, correlations were highest in the grey matter and cerebrospinal fluid (Spearman's $\rho \leq 0.80$), and lower in white matter (Fig. S5A,B). Within regions of interest of the MMP atlases, correlations were lower but predominantly positive, both at the high resolution ($\rho \leq 0.41$; Fig. S5C) and at the low resolution ($\rho \leq 0.30$; Fig. S5D).

We next quantified the within- and between-participant correspondence of EPImix and single-contrast data (Fig. S6A). We calculated global identifiability, as the difference of the median between-participant correlation and median within-participant correlation (Fig. S6B; relevant parts of the correlation matrices are depicted in Fig. S6C). Identifiability was low at the level of brain voxels ($I_{diff} = 0.62 - 0.50 = 0.12$), but considerably higher when correlating cortical GM voxels only ($I_{diff} = 0.47 - 0.23 = 0.24$). Averaging intensities within regions of interest led to increases in both within-participant and between-participant correlations, resulting in decreased identifiability – both for the high-resolution atlas ($I_{diff} = 0.61 - 0.43 = 0.19$) and the low-resolution atlas ($I_{diff} = 0.78 - 0.66 = 0.12$). For regional data, we additionally used a null model relying on spherical "spin" permutation of cortical regions to account for spatial autocorrelation of the data when quantifying spatial correspondence between contrasts. Within the high-resolution atlas, 66/66 = 100% of within-participant correlations survived the FDR-corrected permutation test, compared to 3243/4290 = 75.6% of between-participant correlations. Within the low-resolution atlas, 64/66 = 97.0% of within-participant correlations survived the permutation test, compared to 3029/4290 = 70.6% of between-participant correlations (Fig. S6A). Finally, we calculated individual-level identifiability, as the fraction of times that within-participant scan correlations are higher than between-participant scan correlations, using one of the contrasts as a reference (Fig. S6D). Individual identifiability was highly similar when using EPImix T_1-w scans and T_1-w scans as reference. Individual participants were most identifiable at the level of GM voxels, with high individual identifiability at the level of all brain voxels and regions of the high-resolution atlas as well; regions of the low-resolution atlas led to comparatively lower individual identifiability (Fig. S6D).

To dissect the effect of voxel-wise smoothing on across- and between-participant correspondence as well as identifiability, we repeated a subset of the above analyses after smoothing voxel-wise data using 2, 4, and 6 mm FWHM kernels, and compared results to unsmoothed data (0 mm FWHM below) (Fig. S7). We first inspected the correlation, across participants, of all brain voxels as a function of smoothing kernel size. The effect of smoothing was to reduce correlations; median correlations decreased as a function of smoothing, both within the whole-brain mask (Md(ρ) for: 0 / 2 / 4 / 6 mm FWHM = 0.17 / 0.17 / 0.15 / 0.13), and within the GM mask (Md(ρ) for: 0 / 2 / 4 / 6 mm FWHM = 0.22 / 0.22 / 0.20 / 0.17) (Fig. S7A). We next investigated between-participant correspondence using voxel-wise GM T_1-w intensities, which is the voxel-wise type of data for which identifiability was highest in unsmoothed data ($I_{diff} = 0.24$, compared to $I_{diff} = 0.12$ for all brain voxels). The effect of smoothing was to increase both within-participant and between-participant correlations, but with a greater increase in the latter; resulting in reduced differential identifiability as a function of increasing smoothing kernel size (I_{diff} for: 0 / 2 / 4 / 6 mm FWHM = 0.24 / 0.24 / 0.21 / 0.16; Fig. S7B,C).

Finally, we constructed networks of T_1-w intensity covariance, using both EPImix and single-contrast scans. While single-contrast T_1-w structural covariance networks showed similar hallmarks of organisation to structural covariance networks commonly constructed from regional cortical thickness or grey matter volume data, such as strong long-range inter-hemispheric correlations between homotopic regions, structural covariance networks constructed from EPImix data instead showed high short-range correlations, clustered in frontal cortex; particularly so for regions of the high-resolution atlas (Fig. S8). The correspondence between the upper triangular parts of the structural covariance matrices was modest for the high-resolution atlas (Spearman's $\rho = 0.22$), with higher correspondence for the low-resolution atlas (Spearman's $\rho = 0.45$).

Step	Evaluation	processing time (s) Δ_{Md}	processing time (s) $\mathbf{P}_{raw}$	processing time (s) $\mathbf{P}_{Bonf.}$	quality (Dice) Δ_{Md}	quality (Dice) $\mathbf{P}_{raw}$	quality (Dice) $\mathbf{P}_{Bonf.}$
**spatial resolution**	1mm SyN | 2mm SyN	-111	$<10^{-10}$	$<10^{-10}$	-0.024	$<10^{-10}$	$<10^{-10}$
	2mm SyN | 3mm SyN	-11	$<10^{-10}$	$<10^{-10}$	-0.028	$<10^{-10}$	$<10^{-10}$
**bias field correction**	2mm SyN | 2mm N4 SyN	6	$<10^{-10}$	$<10^{-10}$	0.011	$<10^{-10}$	$5.7 \cdot 10^{-10}$
**brain extraction**	2mm N4 SyN | 2mm N4 BET SyN	-3	$<10^{-10}$	$<10^{-10}$	-0.0020	0.73	1
**b-spline registration**	2mm N4 SyN | 2mm N4 spl-SyN	17	$<10^{-10}$	$<10^{-10}$	0.00032	0.50	1
**reference pipeline**	2mm N4 SyN | 1mm N4 "slow SyN"	2366	$<10^{-10}$	$<10^{-10}$	0.016	$<10^{-10}$	$3.8 \cdot 10^{-10}$

Table S1: Statistical details of the impact of processing steps on time and quality. For each pair of pipelines under comparison, we list the median within-participant difference in processing time, the median within-participant difference in quality (evaluated using the Dice coefficient), as well as the corresponding Wilcoxon signed-rank test raw P-value and Bonferroni-corrected P-value. Median differences in processing time and quality were calculated by subtracting values corresponding to the first pipeline from the second (within participants); i.e. for Evaluation "A | B", $\Delta_{Md} = Md(\mathrm{B} - \mathrm{A})$.

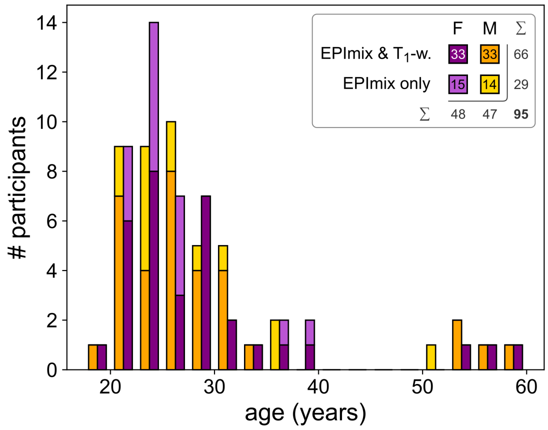

Figure S1: Age distribution of participants by sex and scan sequence. Scans from a total of 95 participants (48 female, 47 male) were included in this study. Of those, 66 (33 female, 33 male) were scanned using both EPImix and single-contrast T_1-weighted sequences, while an additional 29 (15 female, 14 male) were scanned using EPImix only. There were no significant differences in participant age by sex or scan sequence (Chi-squared test, $\chi^2 = 0.005$, P = 0.95).

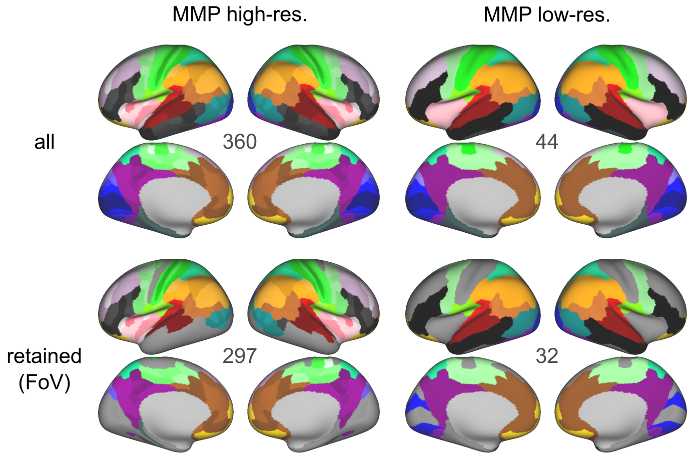

Figure S2: Multi-modal parcellation (MMP) atlases used. Top row: A multi-modal cortical atlas was used at two different spatial resolutions: a high-resolution version (Glasser et al., 2016) and a low-resolution version, created by downsampling continguous regions withing the high-resolution atlas as described in Glasser et al., 2016 SI. Bottom row: Only regions with at least 80% EPImix scan coverage in at least 80% (76/95) participants were used for further analysis. Numbers within each panel correspond to the number of regions in each atlas version.

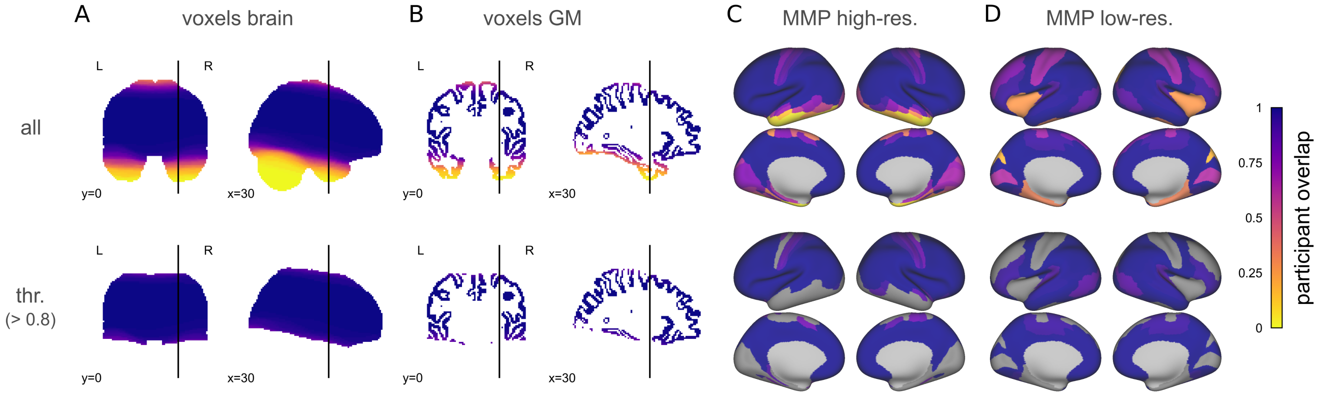

Figure S3: Participant overlap at voxels and regions of interest in EPImix scans with reduced FoV. Top row: Proportion of participants with data at each voxel of A) the (MNI) brain, and B) cortical grey matter (GM). The map in panel B (top) was used to calculate participant overlap within regions of interest, for both C) the high-resolution MMP atlas, and D) the low-resolution MMP atlas. In panels C and D, regions are color-coded by the proportion of participants with at least 80% (non-zero) voxels in each region. Subsequent analyses were limited to voxels and regions with at least 80% participant overlap (bottom row).

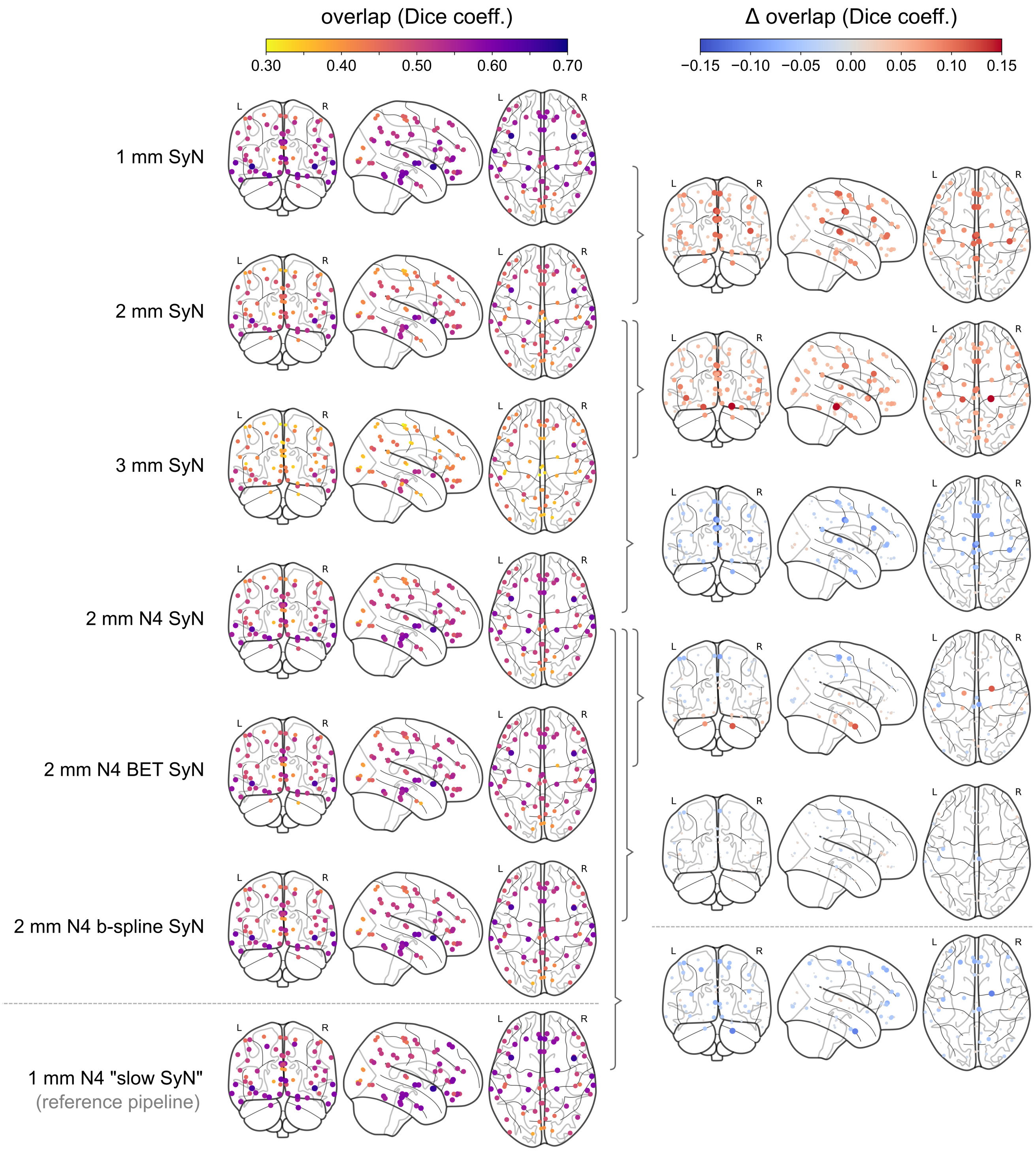

Figure S4: Evaluation of regional quality of registration (and preceding steps) using the Mindboggle dataset. Left: Regional Dice coefficient values quantifying the overlap between "manually" registered atlas labels and those released with the Mindboggle dataset (Klein and Tourville, 2012), for each of seven evaluated processing pipelines (rows 1-3: spatial resolution; row 4: bias field correction; row 5: brain extraction; row 6: b-spline SyN registration; row 7: "reference pipeline" with a slower but higher quality version of the ANTs SyN registration algorithm). Right: Regional differences in Dice coefficient values. Pairs of maps being compared are joined by grey braces. Differences were calculated by subtracting values of the map below from the map above (i.e. $\Delta\text{Dice} = \text{Dice}_{above} - \text{Dice}_{below}$).

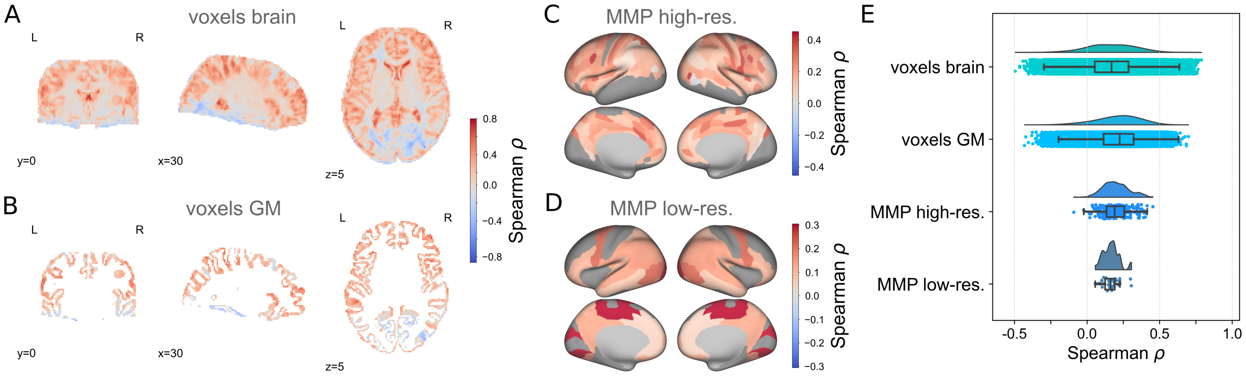

Figure S5: Local correspondence of T_1-w intensities across participants. Spearman's correlations between intensities of rapidly-processed T_1-w scans from the EPImix sequence and a single-contrast acquisition, using data of 66 participants. Correlations are depicted: at the voxel level for A) the whole brain, and B) cortical grey matter, as well as within regions of interest of C) the high-resolution and D) the low-resolution MMP atlas. E) Distributions of correlations at each spatial resolution considered (as depicted in panels A-D). Note that few of these correlations were significant (respectively 20 / 31% of brain / GM voxels, and 3 / 0% regions of the high / low-resolution MMP atlases.) (At the regional level, median regional values were extracted prior to calculation of correlations for each region.)

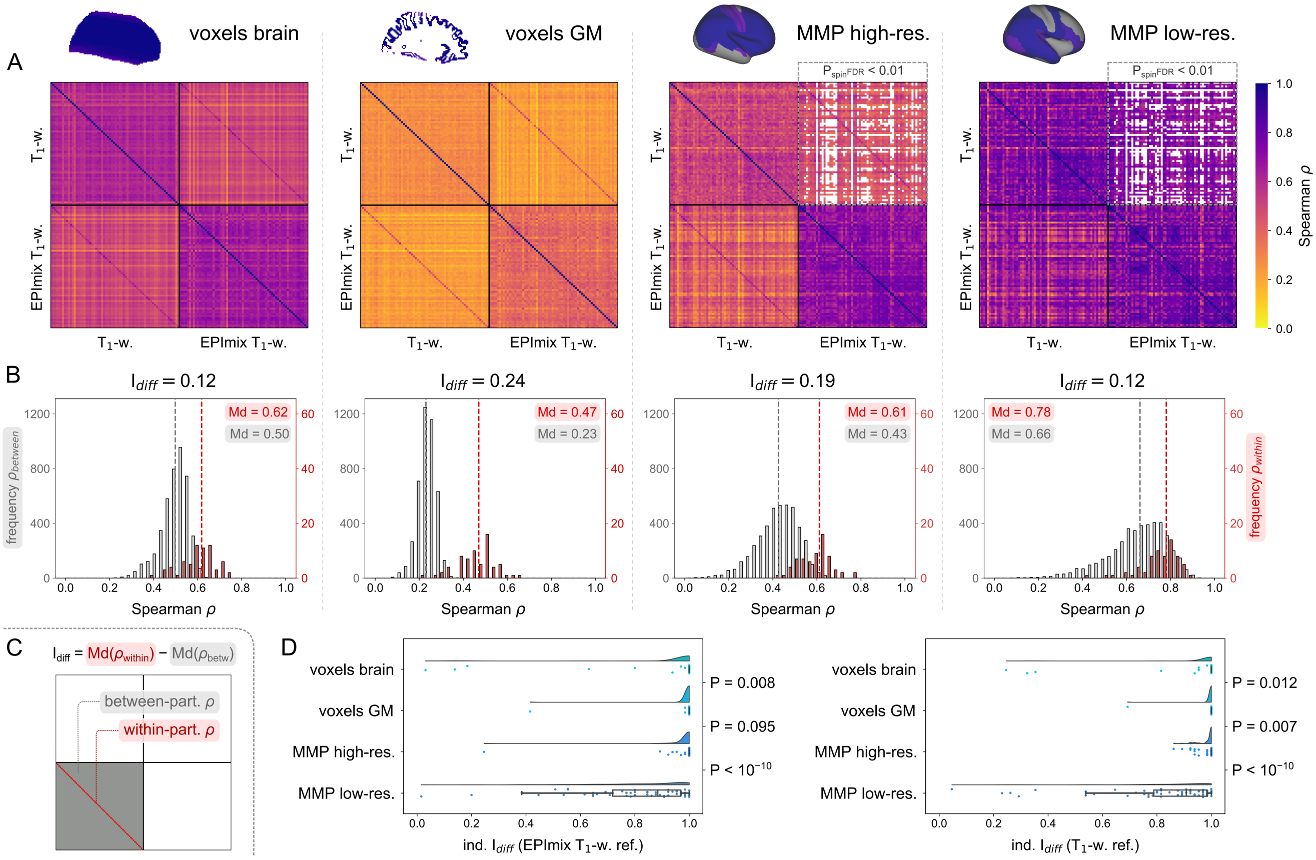

Figure S6: Participant identifiability across EPImix and single-contrast scans, using T_1-w scan intensities. Between-participant correlations and identifiability were investigated using four types of input data, at three spatial resolutions (columns in panels A-B, rows in panel D): all brain voxels, cortical grey matter (GM) voxels, regions of the high-resolution MMP atlas, and regions of the low-resolution MMP atlas. A) Spearman's correlations between EPImix and single-contrast T_1-w scan intensities, within and between participants. Cross-contrast correlations at the level of regions of interest were benchmarked using a null model controlling for contiguity and spatial autocorrelation (upper triangular blocks). B) Differential identifiability of contrasts, defined as the difference between the median within-participant correlation (right / red y-axes) and the median between-participant correlation (left / grey y-axes), as illustrated in C). D) Individual identifiability, defined as the fraction of times that the within-participant correlation is higher than between-participant correlations, either identifying a single-contrast T_1-w scan relative to EPImix T_1-w scans (EPImix T_1-w ref.), or vice-versa (T_1-w ref.). P-values correspond to the (paired) Wilcoxon signed-rank test between neighbouring distributions (testing whether different spatial resolutions of data lead to differences in individual identifiability (H_1), or whether there is no statistical difference between these values (H_0)).

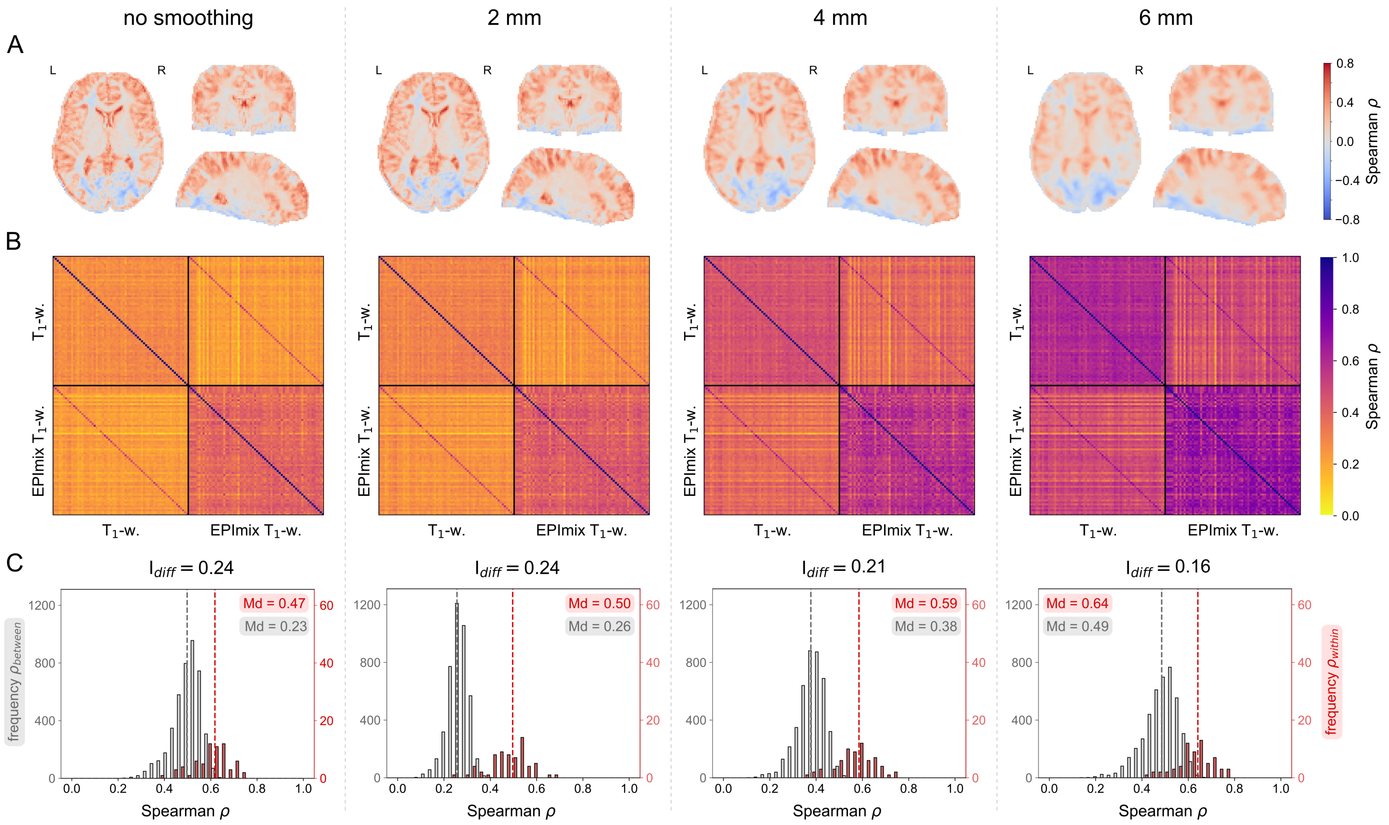

Figure S7: Effects of data smoothing on between-participant correspondence and identifiability of voxel-wise GM T_1-w intensities. A) Spearman's correlations between voxel-wise T_1-w intensities of rapidly-processed scans from the EPImix sequence and a single-contrast acquisition across 66 participants, as a function of smoothing. B) Spearman's correlations between EPImix and single-contrast T_1-w scan intensities, within and between participants. C) Differential identifiability of contrasts, defined as the difference between the median within-participant correlation (right / red y-axes) and the median between-participant correlation (left / grey y-axes) (as illustrated in main text Fig. 5C and Fig S6C).

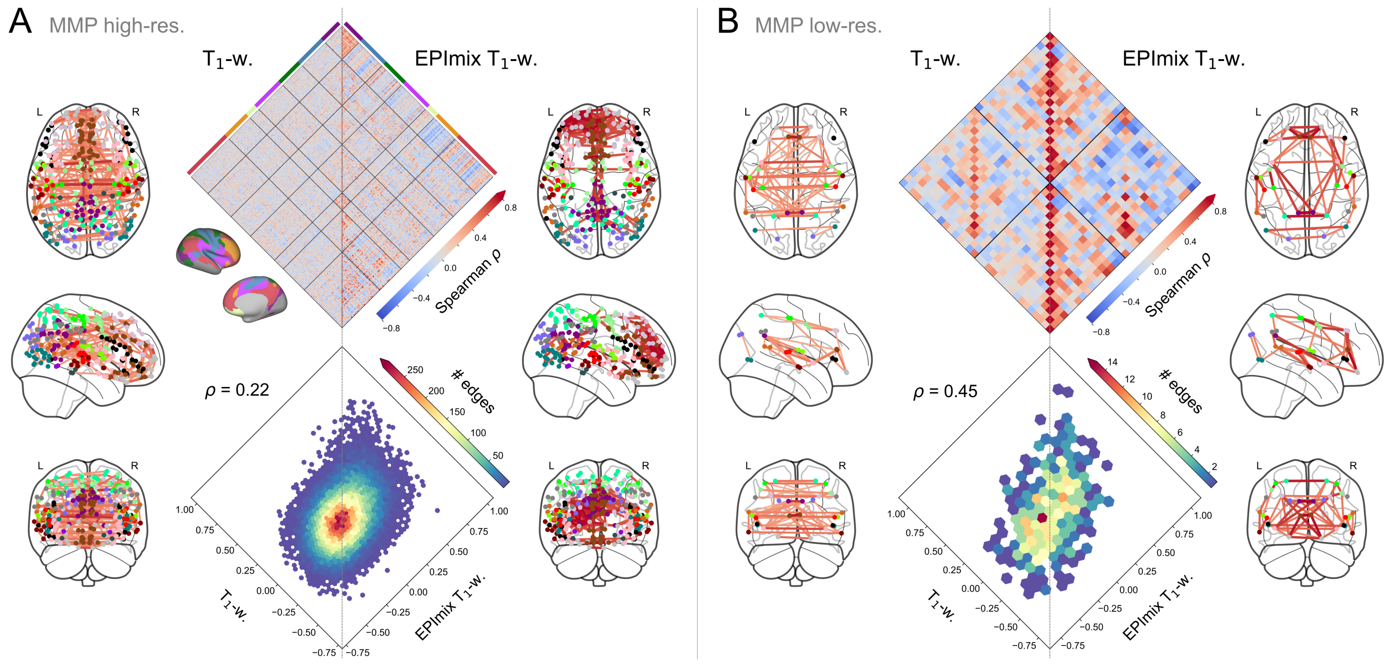

Figure S8: Structural covariance networks constructed from EPImix and single-contrast T_1-w intensities. A) Structural covariance networks constructed using the high-resolution MMP atlas (297 regions). The diamond plot (top) is ordered according to regional membership of the 7 canonical intrinsic connectivity networks derived by Yeo et al., 2011. Network diagrams depict the strongest 0.3% correlations. B) Structural covariance networks constructed using the low-resolution MMP atlas (32 regions). Network diagrams depict the strongest 10% correlations.

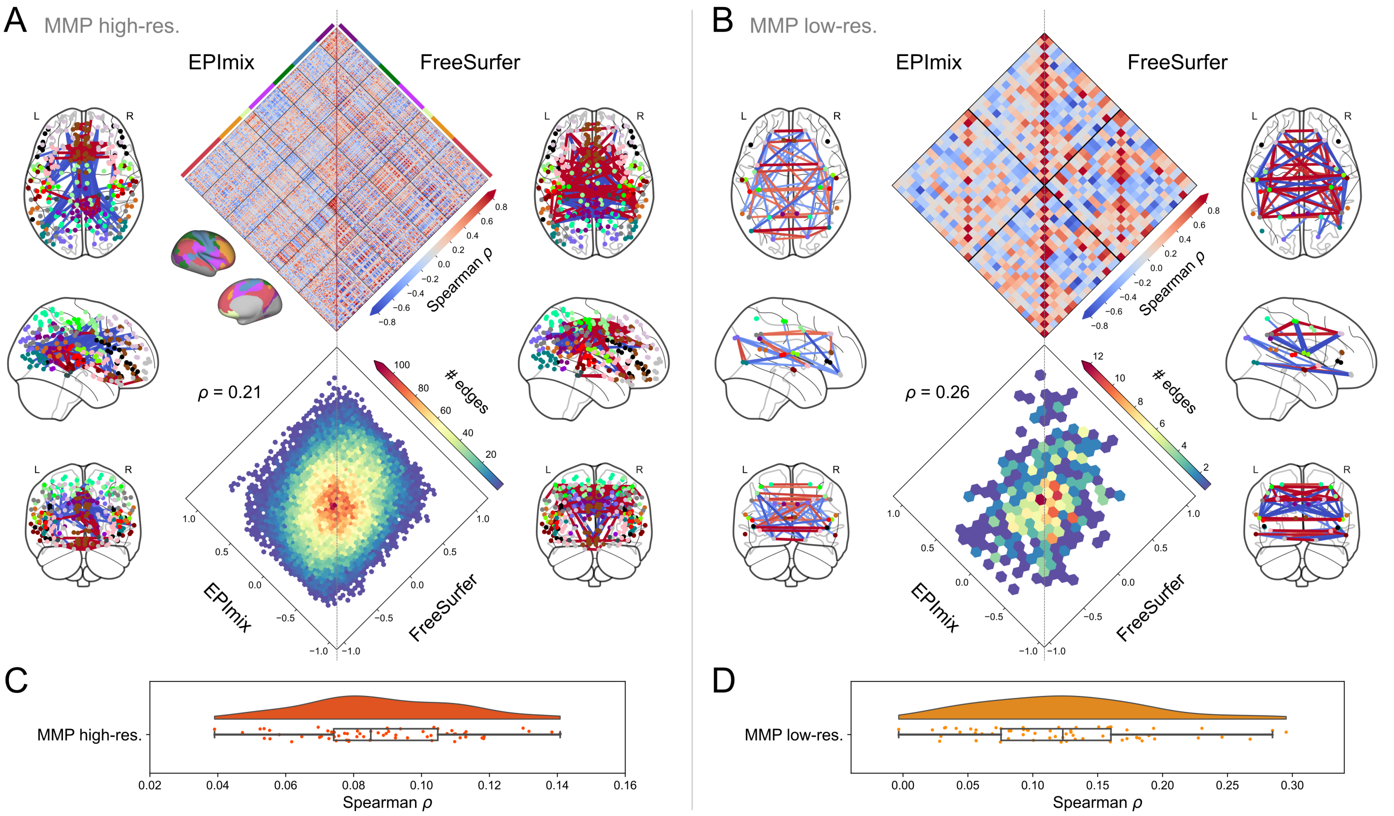

Figure S9: Morphometric similarity networks constructed from EPImix contrasts and FreeSurfer reconstructions of single-contrast T_1-w scans. A) Group-average morphometric similarity networks constructed using the high-resolution MMP atlas (297 regions). The diamond plot (top) is ordered according to regional membership of the 7 canonical intrinsic connectivity networks derived by Yeo et al., 2011. Network diagrams depict the strongest 0.3% correlations. B) Group average morphometric similarity networks constructed using the low-resolution MMP atlas (32 regions). Network diagrams depict the strongest 10% correlations. Panels C) and D) depict the distribution of Spearman's ρ within individual participants.

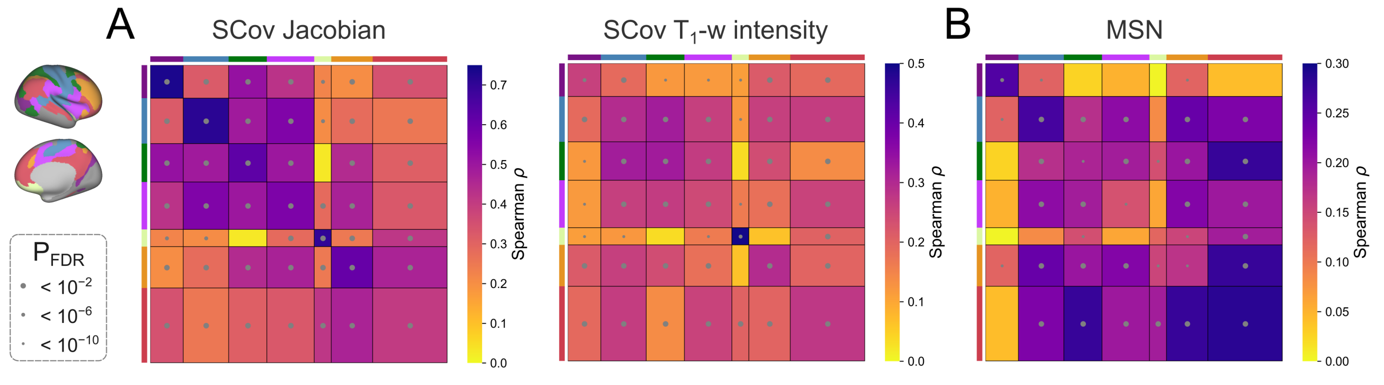

Figure S10: Correspondence between structural covariance and morphometric similarity networks derived from EPImix and single-contrast T_1-w scans across intrinsic connectivity networks. Spearman's ρ between groups of edges within and between intrinsic connectivity networks derived by Yeo et al., 2011, in networks constructed using the high-resolution MMP atlas (297 regions). A) Correlations between structural covariance networks constructed from EPImix and single-contrast T_1-w scan log-Jacobians (left), and T_1-w intensities (right). B) Correlations between morphometric similarity networks constructed from EPImix contrasts and FreeSurfer reconstructions of single-contrast T_1-w scans.

References

Ad-Dab'bagh, Y., Einarson, D., Lyttelton, O., Muehlboeck, J.-S., Mok, K., Ivanov, O., Vincent, R., Lepage, C., Lerch, J., Fombonne, E., and Evans, A. (2006). The CIVET Image-Processing Environment: A Fully Automated Comprehensive Pipeline for Anatomical Neuroimaging Research. In *Proc. 12th Annu. Meet. Organ. Hum. Brain Mapp.*

Alexander-Bloch, A. F., Shou, H., Liu, S., Satterthwaite, T. D., Glahn, D. C., Shinohara, R. T., Vandekar, S. N., and Raznahan, A. (2018). On testing for spatial correspondence between maps of human brain structure and function. *Neuroimage*, 178(February):540–551.

Benjamini, Y. and Hochberg, Y. (1995). Controlling the false discovery rate: a practical and powerful approach to multiple testing.

Glasser, M. F., Coalson, T. S., Robinson, E. C., Hacker, C. D., Harwell, J., Yacoub, E., Ugurbil, K., Andersson, J., Beckmann, C. F., Jenkinson, M., Smith, S. M., and Van Essen, D. C. (2016). A multi-modal parcellation of human cerebral cortex. *Nature*, pages 1–11.

Klein, A. and Tourville, J. (2012). 101 labeled brain images and a consistent human cortical labeling protocol. *Front. Neurosci.*, 6(December):1–12.

Markello, R. D. and Misic, B. (2020). Comparing spatially-constrained null models for parcellated brain maps. *bioRxiv*, pages 1–22.

Skare, S., Sprenger, T., Norbeck, O., Ryd, H., Blomberg, L., Avventi, E., and Engstr, M. (2018). A 1-Minute Full Brain MR Exam Using a Multicontrast EPI Sequence. *Magn. Reson. Med.*, 3054:3045–3054.

Váša, F., Romero-garcia, R., Kitzbichler, M. G., Seidlitz, J., Whitaker, K. J., Vaghi, M. M., Kundu, P., Patel, A. X., Fonagy, P., Dolan, R. J., Jones, P. B., Goodyer, I. M., the NSPN Consortium, Vértes, P. E., and Bullmore, E. T. (2020). Conservative and disruptive modes of adolescent change in human brain functional connectivity. *Proc. Natl. Acad. Sci. U. S. A.*, 117 (6):3248–3253.

Váša, F., Seidlitz, J., Romero-Garcia, R., Whitaker, K., Rosenthal, G., Vértes, P., Shinn, M., Alexander-Bloch, A., Fonagy, P., Dolan, R., Jones, P., Goodyer, I., Sporns, O., and Bullmore, E. (2018). Adolescent tuning of association cortex in human structural brain networks. *Cereb. Cortex*, 28(1).

Yeo, B. T. T., Krienen, F. M., Sepulcre, J., Sabuncu, M. R., Lashkari, D., Hollinshead, M., Roffman, J. L., Smoller, J. W., Zöllei, L., Polimeni, J. R., Fischl, B., Liu, H., and Buckner, R. L. (2011). The organization of the human cerebral cortex estimated by intrinsic functional connectivity. *J. Neurophysiol.*, pages 1125–1165.
